# Neural Network Method of Analysing Sensor Data to Prevent Illegal Cyberattacks

**DOI:** 10.3390/s25175235

**Published:** 2025-08-22

**Authors:** Serhii Vladov, Vladimir Jotsov, Anatoliy Sachenko, Oleksandr Prokudin, Andrii Ostapiuk, Victoria Vysotska

**Affiliations:** 1 Department of Scientific Activity Organization, Kharkiv National University of Internal Affairs, 27, L. Landau Avenue, 61080 Kharkiv, Ukraine; 2 Department of Information Systems and Technologies, University of Library Studies and Information Technologies, 119, Tsarigradsko Shose, 1784 Sofia, Bulgaria; 3 Department of Cybersecurity, International Information Technology University, 34A, Manas Street, Almaty 050000, Kazakhstan; 4 Research Institute for Intelligent Computer Systems, West Ukrainian National University, 11, Lvivska Street, 46009 Ternopil, Ukraine; as@wunu.edu.ua; 5 Department of Teleinformatics, Casimir Pulaski Radom University, 29, Malczewskiego Street, 26-600 Radom, Poland; 6 Department of Organization of Educational and Scientific Training, Kharkiv National University of Internal Affairs, 27, L. Landau Avenue, 61080 Kharkiv, Ukraine; oleksandr.prokudin@ukr.net; 7 Lviv State University of Life Safety, 79000 Lviv, Ukraine; ostapyuk.andriy@ukr.net; 8 Information Systems and Networks Department, Lviv Polytechnic National University, 12, Bandera Street, 79013 Lviv, Ukraine; victoria.a.vysotska@lpnu.ua

**Keywords:** sensor system, sensory data, neural network, loss function, residuals, cyberattacks, cyber police

## Abstract

This article develops a method for analysing sensor data to prevent cyberattacks using a modified LSTM network. This method development is based on the fact that in the context of the rapid increase in sensor devices used in critical infrastructure, it is becoming an urgent task to ensure these systems’ security from various types of attacks, such as data forgery, man-in-the-middle attacks, and denial of service. The method is based on predicting normal system behaviour using a modified LSTM network, which allows for effective prediction of sensor data because the F1 score = 0.90, as well as on analysing anomalies detected through residual values, which makes the method highly sensitive to changes in data. The main result is high accuracy of attack detection (precision = 0.92), achieved through a hybrid approach combining prediction with statistical deviation analysis. During the computational experiment, the developed method demonstrated real-time efficiency with minimal computational costs, providing accuracy up to 92% and recall up to 89%, which is confirmed by high AUC = 0.94 values. These results show that the developed method is effectively protecting critical infrastructure facilities with limited computing resources, which is especially important for cyber police.

## 1. Introduction and Related Works

In recent years, there has been a rapid growth in the number of Internet of Things (IoT) devices [1] and sensor systems [2] integrated into industrial [3,4], consumer [5,6], transport [7,8,9], and critical infrastructure [10,11,12]. These devices collect massive amounts of data in real time, providing environmental monitoring [13], process control [14], and user scenarios [15]. At the same time, the growth in the number of connected sensors creates new vectors for cyberattacks [16,17,18]—attackers can inject malicious packets into sensors, distorting readings or disrupting system operation.

Sensor data have traditionally been considered in the controlled processes context [19,20], but in recent years, they have been actively used to detect anomalies [21] and cyberattacks [22]. Information coming from the network’s different points allows us to identify atypical patterns of behaviour, such as a sudden increase in message frequency [23] or deviations in the physical quantity spectrum [24]. However, classic statistical methods often fail to cope with high dimensionality and non-trivial correlation between data channels.

The relevance of developing a neural network method for analysing sensor data to prevent cyberattacks is determined by a combination of factors: the increasing attack-type complexity (including targeted and hidden), the need to process multidimensional flows in real time, and limited device resources (energy consumption, computing power). Machine learning methods and neural networks [25,26,27,28] will allow adaptation to the object’s changing operating conditions and the detection of new, previously unseen attack signatures.

The first studies on neural network application in information security problems relied on simple fully connected architectures [29] and “shallow learning” [30]. These methods demonstrated satisfactory accuracy (up to 80–85%) on small datasets but were inferior in scalability and sensitivity to noise in the data. Their key limitation was the lack of a built-in feature extraction mechanism, which required careful manual selection and preprocessing.

With the development of deep learning technologies, convolutional neural networks (CNNs) have become widely used for working with sensory data in time series [31,32]. CNNs are good at extracting local patterns and are able to detect temporal anomalies without complex feature engineering. A number of studies, such as [32,33], have shown that convolutional filters are effective in analysing vibration, acoustic, and temperature sensors.

In parallel with CNNs, special attention has been paid to recurrent neural networks (RNNs), in particular, LSTM [34] and GRU [35], due to their ability to take into account long-term dependencies in time series. The developed models have shown high accuracy (over 90%) in detecting gradual changes in the sensor system’s behaviour, median attack characteristics [36], or covert malware injection [37].

Autoencoders [38] and variational autoencoders (VAEs) [39] have laid the foundation for unsupervised anomaly detection; by learning to reconstruct “normal” sensor patterns, they exhibit low reconstruction errors (less than 1–2% [40,41]) when encountering anomalous, potentially malicious signals. These approaches are instrumental in settings where labelled data are scarce.

Recently, graph neural networks (GNNs) have been actively developed for modelling correlations between sensors in distributed systems [42]. By representing a sensor network as a graph, where nodes are sensors and edges are communication channels, GNNs allow spatial and topological dependencies to be taken into account, which increases the accuracy of localisation.

Hybrid methods that combine classic statistical models with neural network blocks [43,44,45] have become popular due to the balance between interpretability and adaptability. In [46], an approach for unsupervised anomaly detection in sensor streams by combining statistical models and deep networks is presented. In [47], a label noise filter ensemble is proposed to improve local diagnostic interpretation of diabetes readings; in [48], an LSTM soft sensor for batch processes with just-in-time multi-model learning is developed; and in [49], a discrete conformal fractional sensing system for predicting CO_2_ emissions is described. However, the approaches considered [46,47,48,49] face a number of limitations: they require a large amount of labelling or threshold fine-tuning, are rarely tested on real data streams with conceptual drift, and often do not take into account the strict limitations of computational resources and the need for interpretability in online monitoring conditions. Thus, architectures where preliminary feature selection is based on signal entropy analysis and subsequent classification is performed using deep neural networks demonstrate better results on multichannel sensor arrays (Table 1).

Despite the successes, there are a number of unsolved problems:

- The lack of unified datasets for evaluating the comparative effectiveness complicates the optimal architecture choice.
- Many studies do not take into account the real transmission delays and end-device resource limitations.
- Neural network solution interpretability. Security operators often require explanations of why a particular point was marked as abnormal.
- Without the deep models’ “black box”, practical implementation in industrial systems is complex.

In addition, when developing methods for analysing sensory data, it is vital to consider resistance to adversarial attacks [50]—attackers can deliberately distort the input signal in such a way that the model will not notice the anomaly or, conversely, will create false positives. Embedding neural network models directly into sensor units (edge computing [51]) requires architecture optimisation in terms of memory and energy consumption, which remains a relevant research area. Thus, the development of a new neural network method for analysing sensory data should solve the following key problems: adaptability to new types of attack, resistance to adversarial disturbances, results interpretability, and the ability to work under conditions of limited computing resources.

## 2. Materials and Methods

### 2.1. Theoretical Foundations of Sensory Data Analysis for Cyberattack Prevention

**Figure 1:**
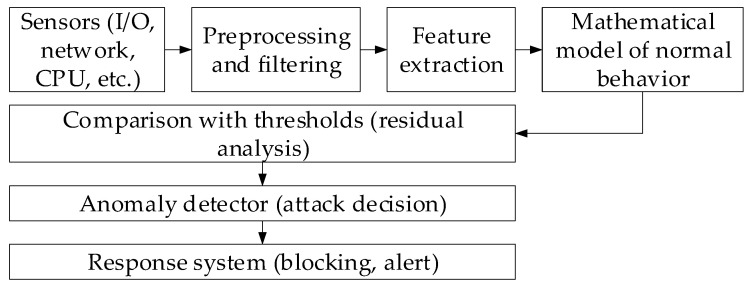
The sensory data analysis method for preventing cyberattacks: a block diagram.

It is known that in complex technical systems operating under modern conditions, sensory data grows exponentially. These flows allow anomalies to be detected and timely analyses associated with cyberattacks performed at early stages [2,16,18]. This study proposes a structure for the analysis of a sensory data method to prevent cyberattacks (Figure 1) that reflects the main stages of processing and detecting such threats.

The sensory data model is based on the fact that **x**(*t*) is a readings vector from *n* sensors at time *t* [18]. It is assumed that in regular operation, the system is described by a linear stochastic differential equation:

(1) $$dx\left(t\right)=A\cdot x\left(t\right)dt+B\cdot dw\left(t\right),$$

where *A* ∈ ℝ*^n^*^×^*^n^* is the normal behaviour dynamics matrix, *B* ∈ ℝ*^n^*^×^*^m^* is the noise intensities matrix, and **w**(*t*) is an *m*-dimensional Wiener process [52].

In discrete time (with step Δ*t*), this gives the following model:

(2) $$x_{k+1}=\Phi \cdot x_{k}+v_{k},\;v_{k}\sim N\left(0,Q\right),$$

where

(3) $$\Phi =\exp A\cdot ∆t,\;Q=\int_{0}^{∆t}\exp\left(A\cdot \tau \right)\cdot B\cdot B^{⊤}\cdot \exp\left(A^{⊤}\cdot \tau \right)d\tau .$$

To minimise the estimated mean square error and obtain an optimal approximation of the actual state in the presence of the dynamics and observations, a recursive Kalman filter is used [53,54], which, at each step, predicts the system state vector based on the previous estimate and then corrects it, taking into account the incoming measurements. The following expressions describe the recursive Kalman filter:

(4) $$\begin{array}{c} {\hat{x}}_{k\left|k-1\right.}=\Phi \cdot {\hat{x}}_{k-1\left|k-1\right.},\;P_{k\left|k-1\right.}=\Phi \cdot P_{k-1\left|k-1\right.}\cdot \Phi^{⊤}+Q,\\ K_{k}=\Phi \cdot P_{k-1\left|k-1\right.}\cdot H^{⊤}\cdot {\left(H\cdot P_{k\left|k-1\right.}\cdot H^{⊤}+R\right)}^{-1},\\ {\hat{x}}_{k\left|k\right.}={\hat{x}}_{k\left|k-1\right.}+K_{k}\cdot \left(y_{k}-H\cdot {\hat{x}}_{k\left|k-1\right.}\right),\;P_{k\left|k\right.}=\left(I-K_{k}\cdot H\right)\cdot P_{k\left|k-1\right.}, \end{array}$$

where **y***_k_* is the measurement vector (**x***_k_* part), *H* is the observation matrix, and *R* is the measurement noise covariance.

The residual (innovation) at the *k*-th step is the difference between the actual measurement and the predicted observed value, which is defined as follows:

(5) $$r_{k}=y_{k}-H\cdot {\hat{x}}_{k\left|k-1\right.},$$

and characterises the deviation magnitude from the model; with normal behaviour, **r***_k_* ∼ *N*(0, *S_k_*) has a zero mean and a covariance of the following form:

(6) $$S_{k}=H\cdot P_{k-1\left|k-1\right.}\cdot H^{⊤}+R.$$

To reliably detect deviations from the system’s normal behaviour, a statistical test is constructed based on residuals **r***_k_*. The key idea is to compare the “distance” between the actual measurements and the predicted state with a threshold that ensures a false-positive given level. For this, the statistics of the form are defined as follows:

(7) $$d_{k}=r_{k}^{⊤}\cdot S_{k}^{-1}\cdot r_{k}.$$

Under the hypothesis “no attack” (*H*_0_), **r***_k_* ∼ *N*(0, *S_k_*) holds, whence $d_{k}\sim \chi_{m}^{2}$, where *m* = rank(*S_k_*), usually equal to the measurement vector dimension. The threshold *γ* is selected so that the exceeding probability during normal behaviour does not exceed a predetermined level α (the false alarm level):

(8) $$P_{FA}=P\left(d_{k}>\gamma |H_{0}\right)=\int_{\gamma }^{\infty }f_{\chi_{m}^{2}}\left(x\right)dx=\alpha .$$

From the inverse distribution function *χ*^2^, we obtain the following:

(9) $$\gamma =F_{\chi_{m}^{2}}^{-1}\left(1-\alpha \right).$$

For example, for *m* = 3 and *α* = 0.01, the threshold is *γ* ≈ 11.34.

In the presence of an attack (hypothesis *H*_1_), the residuals acquire a nonzero mathematical expectation:

(10) $$r_{k}\sim N\left(\mu_{r},S_{k}\right),\;\mu_{r}=H\cdot \left(\Phi \cdot {\hat{x}}_{k-1\left|k-1\right.}+U\cdot u_{0}\right)-H\cdot \Phi \cdot {\hat{x}}_{k-1\left|k-1\right.}=H\cdot U\cdot u_{0}.$$

Then, *d_k_* has a nonparametric distribution (of a nonzero quadratic form):

(11) $$d_{k}\sim \chi_{m}^{2}\left(\lambda \right),\;\lambda =\mu_{r}^{⊤}\cdot S_{k}^{-1}\cdot \mu_{r},$$

that is, the unstandardised *χ*^2^ distribution with the uncentered parameter *λ* [55]. Then, the detection probability is represented as follows:

(12) $$P_{D}=P\left(d_{k}>\gamma |H_{1}\right)=1-F_{\chi_{m\left(\lambda \right)}^{2}}\left(\gamma \right).$$

The *λ* value is proportional to the attack $u_{0}^{⊤}\cdot U^{⊤}\cdot S_{k}^{-1}\cdot H\cdot U\cdot u_{0}$ “strength”.

In the sequential control scheme (every frame *k*), the detection time is introduced:

(13) $$T=\min\left\{k\geq 1:d_{k}>\gamma \right\}.$$

For a small α and constant noise drift, it can be shown through the unquenched *χ*^2^ additivity property that

(14) $$E\left[T\right]\approx \frac{\gamma -m}{\lambda }.$$

It follows from the average E[*d_k_*] ≈ *m* + *k* ∙ *λ* uniform increment and the expectation of the line’s growth rate.

Thus, the threshold *γ* choice determines a trade-off between the false positive rate and the missing attacks probability, while the unstandardised *χ*^2^ distribution allows us to express the PD test explicitly in terms of the uncentered parameter *λ*, and the average detection delay time is estimated as approximately $\frac{\gamma -m}{\lambda }$. This allowed us to formulate Theorem 1, “Detection Time Theorem”:

**Theorem 1.** 
*Let the attack introduce a constant offset u_0_ ≠ 0 into the system with the described model, and let the detector be tuned to a threshold γ corresponding to the false positive rate α. Then, the detection time T = min{k ≥ 1: d_k_ > γ} satisfies $E\left[T\right]\leq \frac{\gamma -m}{u_{0}^{⊤}\cdot W\cdot u_{0}}$.*

**Proof of Theorem 1.** 
The proof of this theorem consists of three steps:

- Step 1. The *d_k_* statistic expectation growth.
- Step 2. Markov estimate of the time to reach the level.
- Step 3. The average E[*T*] estimation.

To estimate the *d_k_* statistic expectation growth, the introduced statistic $d_{k}=r_{k}^{⊤}\cdot S_{k}^{-1}\cdot r_{k}$ is adopted, which helps the comparison of the “distance” between the actual measurements and the predicted state, with the threshold that provides a false-positive given level. In this case, in the presence of an attack, the residual model has the form $r_{k}=H\cdot U\cdot u_{0}+{\tilde{r}}_{k}$, where ${\tilde{r}}_{k}\sim N\left(0,S_{k}\right)$ is the noise part. Then,

(15) $$E\left[d_{k}\right]=E\left[{\left(H\cdot U\cdot u_{0}+{\tilde{r}}_{k}\right)}^{⊤}\cdot S_{k}^{-1}\cdot \left(H\cdot U\cdot u_{0}+{\tilde{r}}_{k}\right)\right].$$

Expanding the brackets and taking into account that $E\left[{\tilde{r}}_{k}\right]=0$ and $E\left[{\tilde{r}}_{k}\cdot {\tilde{r}}_{k}^{⊤}\right]=S_{k}$, we obtain

(16) $$E\left[d_{k}\right]=u_{0}^{⊤}\cdot U^{⊤}\cdot H^{⊤}\cdot S_{k}^{-1}\cdot H\cdot U\cdot u_{0}+tr\left(S_{k}^{-1}\cdot S_{k}\right)=\lambda +m,$$

where

(17) $$\begin{array}{c} \lambda =u_{0}^{⊤}\cdot W\cdot u_{0},\\ W=U^{⊤}\cdot H^{⊤}\cdot S_{k}^{-1}\cdot H\cdot U,\\ m=rank\left(S_{k}\right). \end{array}$$

Note that in the steady state, *S_k_* → *P*_∞_, and *W* are constant; therefore,

(18) $$E\left[d_{k}\right]\approx m+k\cdot \lambda .$$

At the Markov estimation of the time stage to reach the level, it is assumed that *T* is the threshold *γ* first-crossing time. Then, for any *N* ∈ ℕ,

(19) $$\left\{T>N\right\}⟹d_{1}\leq \gamma ,\ldots d_{N}\leq \gamma ⟹\sum_{k=1}^{N}d_{k}\leq N\cdot \gamma .$$

By Markov’s inequality for a non-negative random variable $\sum_{k=1}^{N}d_{k}$

(20) $$P\left(T>N\right)=P\left(\sum_{k=1}^{N}d_{k}\leq N\cdot \gamma \right)\leq \frac{E\left[\sum_{k=1}^{N}d_{k}\right]}{N\cdot \gamma }\leq \frac{\sum_{k=1}^{N}\left(m+k\cdot \lambda \right)}{N\cdot \gamma }.$$

The arithmetic progression sum gives

(21) $$\sum_{k=1}^{N}\left(m+k\cdot \lambda \right)=N\cdot m+\lambda \cdot \frac{N\cdot \left(N+1\right)}{2}=N\cdot \left(m+\frac{\lambda }{2}\cdot \left(N+1\right)\right).$$

This is why

(22) $$P\left(T>N\right)\leq \frac{m+\frac{\lambda }{2}\cdot \left(N+1\right)}{\gamma }.$$

At the average E[*T*] estimating stage, the classic equality for non-negative integer *T* is used:

(23) $$E\left[T\right]=\sum_{N=0}^{\infty }P\left(T>N\right).$$

Taking into account the *P*(*T* > *N*) assessment,

(24) $$E\left[T\right]\leq \sum_{N=0}^{\infty }\frac{m+\frac{\lambda }{2}\cdot \left(N+1\right)}{\gamma }=\frac{1}{\gamma }\cdot \sum_{N=0}^{\infty }\left(m+\frac{\lambda }{2}\cdot \left(N+1\right)\right).$$

However, series $\sum_{N=0}^{\infty }\left(N+1\right)$ diverges, so we truncate the sum before the first intersection, approximating the expected detection time T*, at which the average $E\left[d_{T^{*}}\right]=\gamma$. From the first step

(25) $$m+T^{*}\cdot \lambda =\gamma ⟹T^{*}=\frac{\gamma -m}{\lambda }.$$

Based on the inequality for the first level crossing time, an estimate was obtained:

(26) $$E\left[T\right]\leq \frac{\gamma -m}{\lambda }\leq \frac{\gamma -m}{u_{0}^{⊤}\cdot W\cdot u_{0}}.$$

Thus, the detection time is inversely proportional to the attack “strength” $u_{0}^{⊤}\cdot W\cdot u_{0}$ square and linearly depends on the chosen threshold *γ*. The theorem is proved. □

### 2.2. Development of a Neural Network Method for Analysing Sensory Data to Prevent Cyberattacks

**Figure 2:**
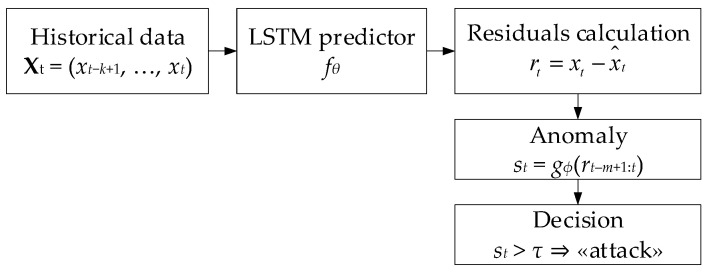
The proposed method’s architectural scheme.

Based on the theoretical model and residual statistics, the study proposes a hybrid method (Figure 2) that consists of training a neural network predictor of normal behaviour and an anomaly classification component based on residuals.

The LSTM predictor *f_θ_* takes as input a sliding window of *k* − 1 previous samples {*x_t_*_−*k*+1_, …, *x_t_*_−1_} and passes them through several LSTM layers [21,34,56] to capture both short-term and long-term dependencies, after which a linear layer produces a one-step prediction ${\hat{x}}_{t}$. In the next step, the residual $r_{t}=x_{t}-{\hat{x}}_{t}$ is calculated, and the matrix *R_t_* = [*r_t_*_−*m*+1_, …, *r_t_*] is formed from the last *m* residual, which is fed into the anomaly classifier input *g_ϕ_* (e.g., a feedforward neural network) trained on normal and synthetically distorted data, after which a scalar score *s_t_* is obtained. Threshold *τ* is chosen based on the validation set so that the *P_FA_* false-positive proportion does not exceed a predetermined level, which ensures the required balance between the detector’s sensitivity and specificity.

**Figure 3:**
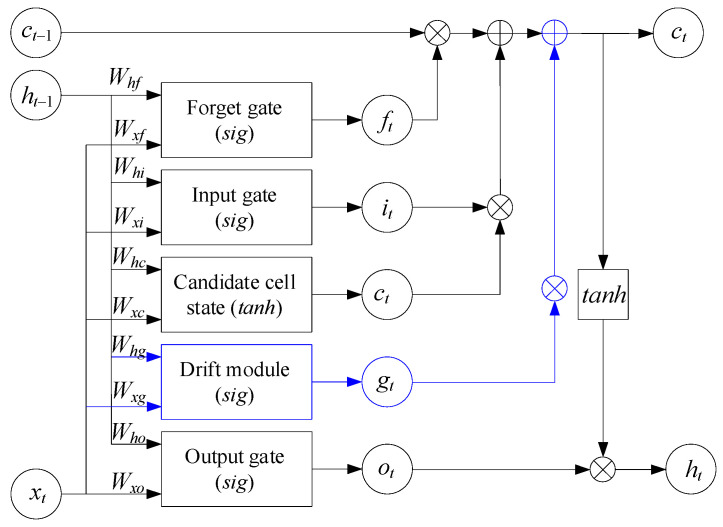
The LSTM predictor architecture uses a modified LSTM cell.

The LSTM predictor (Figure 3) is a modified standard LSTM cell with the addition of “module drift”, which is an additional gate *d_t_*, which introduces adaptive drift into the cell state. The following parameters describe the LSTM predictor: *x_t_* ∈ ℝ*^n^* is the input vector at time *t*, *h_t_*_−1_ ∈ ℝ*^h^* is the previous hidden state and cell state, W* and U* are weight matrices, b* represents biases for the corresponding gates, *σ*(●) is the sigmoid, ⊙ is element-wise multiplication, and tanh(●) is the hyperbolic tangent.

The LSTM cell’s classic gates (input gate, forget gate, output gate, and state candidate) are described by traditional expressions [21,34,56]:

(27) $$\begin{array}{c} i_{t}=\sigma \left(W_{i}\cdot x_{t}+U_{i}\cdot h_{t-1}+b_{i}\right),\\ f_{t}=\sigma \left(W_{f}\cdot x_{t}+U_{f}\cdot h_{t-1}+b_{f}\right),\\ o_{t}=\sigma \left(W_{o}\cdot x_{t}+U_{o}\cdot h_{t-1}+b_{o}\right),\\ {\tilde{c}}_{t}=tanh\left(W_{c}\cdot x_{t}+U_{c}\cdot h_{t-1}+b_{c}\right). \end{array}$$

The proposed drift module introduces an additional gate dt into the LSTM cell, which adaptively regulates the special “drift” vector influence on the state update, taking into account both the current input and the previous hidden state. It allows the model to adjust predictions in the sensory data, gradually changing the base-level context according to the following expression:

(28) $$d_{t}=\sigma \left(W_{d}\cdot x_{t}+U_{d}\cdot h_{t-1}+b_{d}\right),$$

where *W_d_* ∈ ℝ*^h^*^×*n*^, *U_d_* ∈ ℝ*^h^*^×*h*^, *b_d_* ∈ ℝ*^h^*.

The drift signal can be taken either as a constant vector *u*_0_ or as a previous state function:

(29) $$\delta_{t}=V_{d}\cdot h_{t-1}+c_{d},$$

where *V_d_* ∈ ℝ*^h^*^×*h*^, *c_d_* ∈ ℝ*^h^*. Then, the “drift contribution” to the cell state is carried out according to the following expression:

(30) $$∆c_{t}=d_{t}⊙\delta_{t},$$

The cell state update combines the previous state forgetting effects, adding new information and adaptive drift through the corresponding gates:

(31) $$c_{t}=f_{t}⊙c_{t-1}+i_{t}⊙{\tilde{c}}_{t}+d_{t}⊙\delta_{t}.$$

The hidden state *h_t_* is obtained by modulating the cell state activation function output gate-filtered value:

(32) $$h_{t}=o_{t}⊙tanh\left(c_{t}\right).$$

To obtain a one-step prediction after *L* cells, the hidden state *h_t_* is passed through a linear output layer:

(33) $${\hat{x}}_{t+1}=W_{y}\cdot h_{t}+b_{y},$$

where *W_y_* ∈ ℝ*^n^*^×*h*^, *b_y_* ∈ ℝ*^n^*.

Thus, the proposed extension allows the LSTM model not only to take into account the input and past states but also to adaptively add predictions taking into account drift, which allows the slowly changing underlying mode of sensory data to be considered.

The loss function for training the LSTM predictor *f_θ_* is constructed based on the mean square error of predictions and weight regularisation [21,34,56], which is formalised as follows. Let there be a training dataset of length *T* sequences, and for each step *t* = *k*, …, *T* − 1, an input window **X**_t_ = (*x_t_*_−*k*+1_, …, *x_t_*) is formed and the vector ${\hat{x}}_{t+1}=f_{\theta }\left(X_{t}\right)$ prediction is made. Then, the empirical loss function is defined as follows:

(34) $$L_{pred}\left(\theta \right)=\frac{1}{T-k}\cdot \sum_{t-k}^{T-1}{\left\|x_{t+1}-f_{\theta }\left(x_{t-k+1},\ldots ,x_{t}\right)\right\|}_{2}^{2}+\lambda_{\theta }{\left\|\theta \right\|}_{2}^{2},$$

where ${\left\|x_{t+1}-{\hat{x}}_{t+1}\right\|}_{2}^{2}$ is the squared prediction error, the coefficient $\frac{1}{T-k}$ ensures normalisation by the step number, and ${\left\|\theta \right\|}_{2}^{2}=\sum_{i}\theta_{i}^{2}-L^{2}$ is the weight decay regularisation controlled by hyperparameter *λ_θ_* > 0, which prevents overfitting and guarantees the solution’s smoothness.

In the optimisation statement, this function’s mathematical expectation is minimised over the training data distribution [57]:

(35) $$\theta^{*}=\arg\;\underset{\theta }{\min}\;E_{train}\left[{\left\|x_{t+1}-f_{\theta }\left(X_{t}\right)\right\|}_{2}^{2}\right]+\lambda_{\theta }{\left\|\theta \right\|}_{2}^{2}.$$

For numerical optimisation, mini-batch stochastic gradient descent (SGD) [58] is usually used, in which at each *j*-th step, the parameter update is given by the following rule:

(36) $$\theta^{j+1}=\theta^{j}-\eta \cdot \left({𝛻}_{\theta }L_{pred}^{B_{j}}\left(\theta^{j}\right)\right),$$

where *η* is the learning rate and $L_{pred}^{B_{j}}$ is the loss function averaged over batch *B_j_*. In this case, the stochastic gradient descent mini-batch adaptive versions are allowed to be used in the Adam or RMSProp optimiser form [59].

To improve the process time structure reproduction quality, it is proposed that the term $\mu \cdot \sum_{t=k}^{T-2}{\left\|\left({\hat{x}}_{t+2}-{\hat{x}}_{t+1}\right)-\left(x_{t+2}-x_{t+1}\right)\right\|}_{2}^{2}$ (where the coefficient *μ* > 0 specifies this penalty weight) is added to the loss function, penalising the discrepancy between the predicted and actual data change rates, which ensures the prediction’s “smoothness”.

Taking into account the added penalty for the prediction’s “smoothness”, the resulting loss function is represented as follows:

(37) $$L_{pred}\left(\theta \right)=\underset{MSE\;predictions}{\underbrace{\frac{1}{T-k}\cdot \sum_{t-k}^{T-1}{\left\|x_{t+1}-f_{\theta }\left(x_{t-k+1},\ldots ,x_{t}\right)\right\|}_{2}^{2}+\lambda_{\theta }{\left\|\theta \right\|}_{2}^{2}}}+\mu \cdot \underset{Penalty\;for\;the\;dynamics\;inconsistency\;}{\underbrace{\sum_{t=k}^{T-2}{\left\|\left({\hat{x}}_{t+2}-{\hat{x}}_{t+1}\right)-\left(x_{t+2}-x_{t+1}\right)\right\|}_{2}^{2}}}$$

To describe the residuals and the classifier input, it is assumed that at each moment *t*, the actual sensory data values $x_{t}={\left(x_{t}^{\left(1\right)},x_{t}^{\left(2\right)},\ldots ,x_{t}^{\left(n\right)}\right)}^{⊤}\in R^{n}$ vector is received and the corresponding prediction ${{\hat{x}}_{t}=f}_{\theta }\left(x_{t-k+1},\ldots ,x_{t}\right)\in R^{n}$ is made. Then, the residual vector is defined as follows:

(38) $$r_{t}=x_{t}-{\hat{x}}_{t}\in R^{n}.$$

To feed information about the residue’s dynamics into the classifier input, a sliding window of length *m* of the form is formed:

(39) $$R_{t}=\left(r_{t-m+1},r_{t-m+2},\ldots ,r_{t}\right)\in R^{n\times m}.$$

In order to take into account the different scales of sensor measurements, the sensor data values are normalised using their empirical covariance *S* and “whitened” by the residuals:

(40) $${\tilde{r}}_{\tau }=S^{-\frac{1}{2}}\cdot r_{\tau },\;{\tilde{R}}_{t}=\left[{\hat{r}}_{t-m+1},{\tilde{r}}_{t-m+2},\ldots ,{\tilde{r}}_{t}\right].$$

To feed the LSTM predictor, the matrix ${\tilde{R}}_{t}$ is “straightened” into a vector of the form

(41) $$z_{t}=vec\left({\tilde{R}}_{t}\right)=\left(\begin{array}{c} {\hat{r}}_{t-m+1}\\ {\tilde{r}}_{t-m+2}\\ \ldots \\ {\tilde{r}}_{t} \end{array}\right)\in R^{n\cdot m},$$

which serves as the anomaly classifier *s_t_* = *g_ϕ_*(*z_t_*) input, which, in turn, based on it, produces a scalar score *s_t_* ∈ [0, 1], interpreted as the anomaly posterior probability.

**Figure 4:**
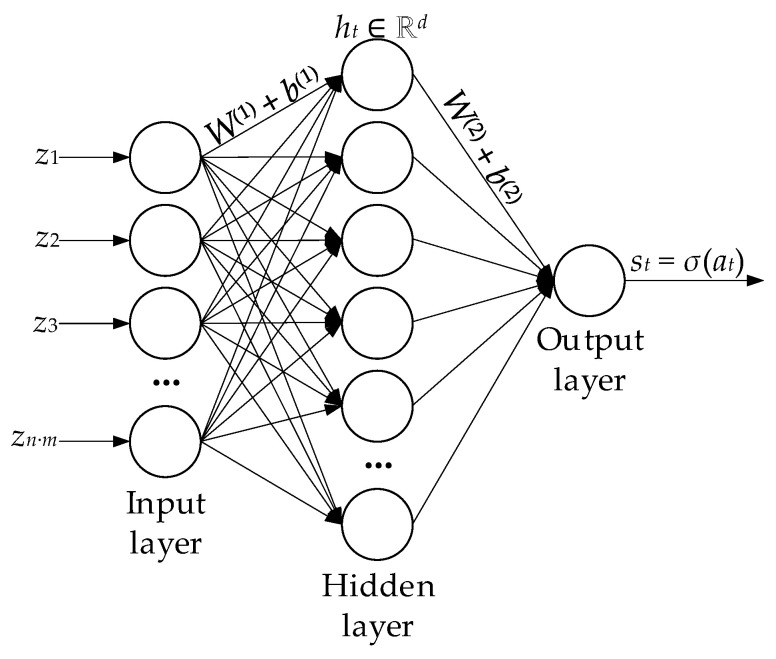
The single-layer MLP detector with one hidden layer architecture diagram.

In this research, a single-layer MLP detector with one hidden layer is used [60,61] (Figure 4), which is described by the following expression for linear transformation and activation:

(42) $$h_{t}=\phi_{1}\left(W^{\left(1\right)}\cdot z_{t}+b^{\left(1\right)}\right)\in R^{d},$$

where *W*^(1)^ ∈ ℝ*^d^*^×(*n* ∙ *m*)^, *b*^(1)^ ∈ ℝ*^d^* are the first layer parameters, *ϕ*_1_(⋅) is the SmoothReLU activation function [62,63], and *d* represents the hidden neurons. The following expressions describe the output layer:

(43) $$a_{t}=W^{\left(2\right)}\cdot z_{t}+b^{\left(2\right)},\;s_{t}=\sigma \left(a_{t}\right)=\frac{1}{1+\exp\left(-a_{t}\right)},$$

where *W*^(2)^ ∈ ℝ^1×*d*^, *b*^(2)^ ∈ ℝ.

According to the logit model derived from the Maxwell–Boltzmann distribution principles [64], the scalar score *s_t_* = *σ*(*a_t_*) is directly interpreted as the posterior probability that the residual’s current window belongs to the anomalous class. That is,

(44) $$\log\left(\frac{P\left(y_{t}=1|z_{t}\right)}{P\left(y_{t}=0|z_{t}\right)}\right)=a_{t},$$

which is why

(45) $$s_{t}=P\left(y_{t}=1|z_{t}\right),\;1-s_{t}=P\left(y_{t}=0|z_{t}\right).$$

The loss function for training the binary anomaly classifier is based on the cross-entropy between the accurate labels *y_t_* and the predicted probabilities *s_t_*, which allows one to directly maximise the correct classification log likelihood, and the weight ${\left\|\phi \right\|}_{2}^{2}$ *L*^2^-regularisation addition prevents overfitting and improves the model’s generalisation ability [21,34]. Thus,

(46) $$L_{clas}\left(\phi \right)=-\frac{1}{N}\cdot \left(y_{t}\cdot \log\left(s_{t}\right)+\left(1-y_{t}\right)\cdot \log\left(1-s_{t}\right)\right)+\lambda_{\phi }\cdot {\left\|\phi \right\|}_{2}^{2}.$$

Gradient descent produces updates of the form:

(47) $$\phi \leftarrow \phi -\eta \cdot {𝛻}_{\phi }L_{clas}\left(\phi \right).$$

The decision rule is based on the obtained scalar score *s_t_* in comparison with a pre-selected threshold *τ*, which is defined in the validation dataset as the value at which the empirical false alarm rate

(48) $${\hat{P}}_{FA}\left(\tau \right)=\frac{1}{N_{0}}\cdot \sum_{y_{t}=0}1\left\{s_{t}>\tau \right\}$$

best corresponds to the required $P_{FA}^{*}$. The decision on “attacks” is made by the threshold. If *s_t_* > *τ*, then a decision is made on the presence of an attack (${\hat{y}}_{t}=1$); otherwise, it remains in the system’s normal state (${\hat{y}}_{t}=0$). Thus,

(49) $${\hat{y}}_{t}=\left\{\begin{array}{c} 1,\;if\;s_{t}>\tau ,\\ 0,\;if\;s_{t}\leq \tau , \end{array}\right.$$

In this case, threshold *τ* is set in such a way as to ensure the required level of false alarms *P_FA_* during validation:

(50) $$\tau =\arg\underset{\tau^{\prime }}{\min}\left\{{\hat{P}}_{FA}\left(\tau^{\prime }\right)-P_{FA}^{*}\right\},$$

where ${\hat{P}}_{FA}\left(\tau^{\prime }\right)$ is determined according to (48).

The anomaly detector quality metrics [65,66,67,68,69] are based on the confusion matrix, which is presented in Table 2.

According to Table 2, four indicators were obtained for *N* tested windows:

(51) $$\begin{array}{c} TP=\sum_{t=1}^{N}1\left\{y_{t}=1,{\hat{y}}_{t}=1\right\},\;FP=\sum_{t=1}^{N}1\left\{y_{t}=0,{\hat{y}}_{t}=1\right\},\\ TN=\sum_{t=1}^{N}1\left\{y_{t}=0,{\hat{y}}_{t}=0\right\},\;FN=\sum_{t=1}^{N}1\left\{y_{t}=1,{\hat{y}}_{t}=0\right\}, \end{array}$$

where *TP* is the number of cases when the model correctly identified an anomaly (the “attack” signal in the presence of a real attack), *TN* is the number of instances where the model correctly identified a normal state (there is no “attack” signal, and there is really no attack), and *FP* is the number of false positives, i.e., the model generated an “attack” signal. In fact, the system was working normally; *FN* is the model’s missed number, i.e., the model did not generate an “attack” signal, although an attack actually occurred.

The correct proportion of detected attacks shows what the real proportion of attacks was that was successfully detected and is defined as follows:

(52) $$Recall=TPR=\frac{TP}{TP+FN}.$$

Precision reflects the correctly recognised proportion of attacks among all “attack” signals and is defined as follows:

(53) $$Precision=\frac{TP}{TP+FP}.$$

The false alarm rate characterises the detector’s tendency to generate false signals and is defined as follows:

(54) $$FPR=\frac{TP}{FP+FN}.$$

The overall recognition accuracy is defined as follows:

(55) $$Accuracy=\frac{TP+TN}{N}.$$

To balance precision and recall, the F-measure (*F_β_*) is used and is defined as follows:

(56) $$F_{\beta }=\left(1+\beta^{2}\right)\cdot \frac{Precision\cdot Recall}{\beta^{2}\cdot \left(Precision+Recall\right)},$$

where *β* > 1 enhances the recall weight (detection value) and *β* < 1 enhances the precision weight.

The *ROC* curve displays the correctly detected proportion of attacks *TPR*(*τ*) in relation to the false alarm *FPR*(*τ*) proportion dependence with varying threshold *τ*, and the area under the *ROC* curve (*AUC*) numerically characterises the model’s generalised ability to separate classes—the closer the *AUC* is to 1, the better the detector distinguishes between normal and abnormal states. The *AUC* is equal to the probability that a randomly selected attack dataset will receive a higher *s_t_* score than a randomly chosen standard dataset. The area under the *ROC* curve (*AUC*) is defined as follows:

(57) $$AUC=\int_{0}^{1}TPR\left({FPR}^{-1}\left(u\right)\right)du$$

and serves as an integral measure of class separability.

The *PR* curve plots the precision on recall dependence when threshold *τ* changes, which is especially important in rare attacks, where the balance between false alarms and missed events is critical. The average precision is calculated as the integral sum of precision over recall increments:

(58) $$PR=\sum_{i=1}^{R}\left({Recall}_{i}-{Recall}_{i-1}\right)\cdot {Precision}_{i},$$

integrating the precision over the recall increments, where *Recall*_0_ = 0 and ${\left\{\left({Precision}_{i},{Recall}_{i}\right)\right\}}_{i=1}^{K}$ are the *PR* curve points, ordered by decreasing speed values.

In addition to traditional metrics, the average response delay is also estimated:

(59) $$E\left[T\right]=\frac{1}{N_{1}}\cdot \sum_{y_{t}=1}\left(t-t_{attack,t}\right),$$

where *t_attack,t_* is the moment the attack begins, and the sum is taken over all windows with real attacks.

To enhance reactivity to prolonged minor deviations, one can construct “sliding” abbreviated sums of speed values:

(60) $$S_{t}=\max\left(0,S_{t-1}+s_{t}-v\right),$$

and signal when *S_t_* > *h*, similar to the CUSUM method [70].

Thus, based on the above, the developed method algorithm was synthesised and is presented in Table 3.

Thus, the developed hybrid approach combines the LSTM predictor’s power, capable of capturing complex nonlinearities, with the classic residuals’ statistics from the SDE model and *χ*^2^ tests, which ensures accurate and provable detection. At the same time, when normal conditions change, it is sufficient to retrain *f_θ_* on new “clean” data, and threshold *τ* is understandably adjusted through the abnormal score *s_t_* empirical distribution in accordance with the false alarm required level *P_FA_*. With weak attacks and correct modelling at the vector *u*_0_ generating stage, a theorem analogous to the average detection delay is easily derived, where the expected increase E[*s_t_*] appears instead of the uncentered parameter *λ*.

## 3. Case Study

### 3.1. Description of the Research Object and Experimental Setup

**Figure 5:**
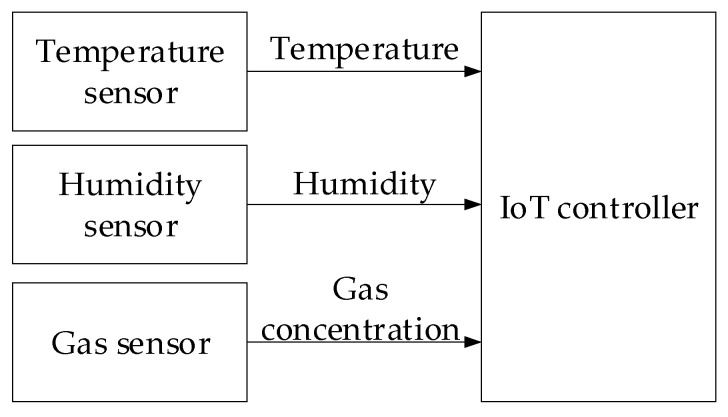
The research object structural diagram.

In this study, the research object is the industrial IoT controller sensor system for monitoring the environment in a government facility server room [71] (Figure 5) where critical equipment is located: servers, network storage, and confidential data processing systems. In this room, it is essential to maintain a stable temperature, optimal humidity, and the absence of harmful gases since even minor deviations can lead to equipment overheating, moisture condensation, or corrosion, which creates the risk of failure of the entire IT infrastructure. The IoT controller consists of three sensors:

- The temperature sensor measures room temperature.
- The humidity sensor measures air humidity.
- The gas sensor measures gas concentrations, such as CO_2_ or volatile organic compounds.

Possible cyberattacks on an IoT controller include the following:

- Spoofing, in which an attacker sends fake numeric values, such as an elevated temperature, to cause an emergency shutdown of the equipment.
- A man-in-the-middle (MITM) attack, which allows the information being transmitted to be intercepted and modified.
- A replay attack, in which old but correct data is transmitted to hide current conditions, and a denial of service (DoS), which disrupts data transmission and paralyses the system.

**Figure 6:**
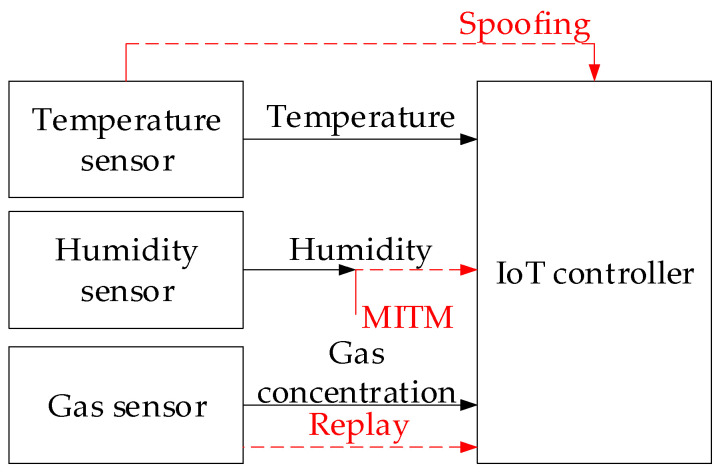
The research object during cyberattacks (spoofing, MITM, Replay) on the IoT controller structural diagram.

Thus, by distorting sensor readings, attackers can cause cooling to be turned off, equipment to overheat, server failures to occur, and, as a result, denial of service (DoS) or loss of access to critical information (Figure 6).

Figure 6 shows how three sensors (temperature, humidity, and gas concentration) transmit key parameters of the server room environment to the IoT controller, while vector cyberattacks are implemented through spoofing (sensor value substitution), MITM (message interception and modification), replay attacks (re-sending old correct data), and DoS (communication channel disruption), which allows attackers to distort or block information and, accordingly, destabilise the entire system operation.

**Figure 7:**
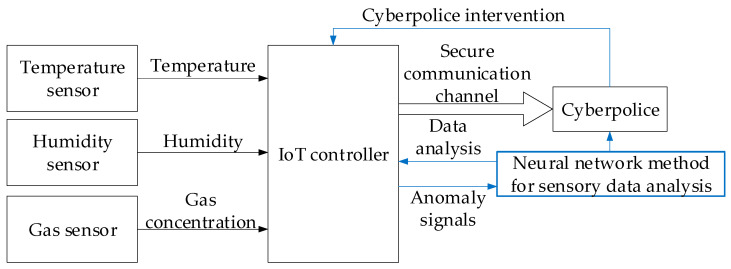
The research object structural diagram takes into account the developed neural network method for analysing sensory data to prevent cyberattacks.

Taking into account the developed neural network method for analysing sensory data presence in a cyber police unit to prevent cyberattacks, the research object structural diagram will be presented in the form of that in Figure 7.

Figure 7 shows how data from three sensors (temperature, humidity, and gas concentration) is received by a local IoT controller and then transmitted via a secure channel to a remote cyber police analytical centre, where a neural network model in real time identifies anomalies characteristic of spoofing, MITM, replay, and DoS attacks and returns alarm signals and recommendations to the controller to block or filter suspicious messages.

**Figure 8:**
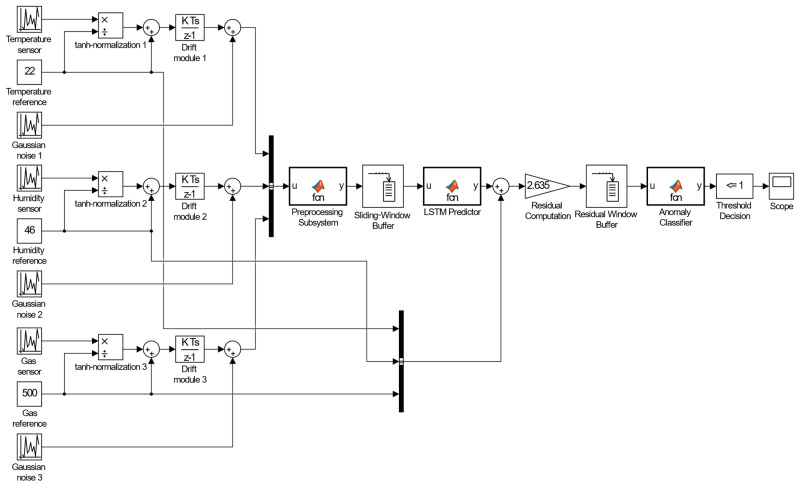
The neural network method for analysing sensory data to prevent cyberattacks was developed, and a test sample scheme was used in the MATLAB Simulink R2014b software environment.

In this research, to conduct a computational experiment, the developed neural network method for analysing sensory data to prevent cyberattacks is implemented as a test sample in the MATLAB Simulink R2014b software environment (Figure 8).

The model subsystems are organised as follows:

- The input sensor data are first normalised in the preprocessing block (tanh transformation taking into account pre-calculated *μ* and *σ*);
- The sliding-window buffer cumulative block forms a vector for the LSTM predictor (MATLAB function) from the previous *k* − 1 sample, which, based on the states *h*(*t* − 1), *c*(*t* − 1), and the model equations, produces a predict $\hat{x}\left(t\right)$ and updated states;
- The Residual Computation block calculates the residual $r_{t}=x_{t}-{\hat{x}}_{t}$ and “whitens” it by multiplying by $S^{-\frac{1}{2}}$;
- The Residual Window Buffer accumulates last *m* vectors *r_whitened_* to form the matrix *R_t_*;
- In the MATLAB Function subsystem, Anomaly Classifier, based on MLP (two linear operations with SmoothReLU and sigmoid), anomaly probability estimate *s*(*t*) is produced for vector *z* = reshape(*R_t_*), which is compared with threshold *τ* in the Threshold Decision (Compare to Constant block) and generates a Boolean alarm signal.

The model’s first block in MATLAB Simulink begins by receiving a three-channel sequential signal (temperature, humidity, and CO_2_) via the Sequence Input block (dimension is 3). Then, the data passes through a sliding-window mechanism based on the Buffer block (window length is 50 samples, step is five samples, and window intersection is 90%), after which it enters two LSTM layers:

- The first LSTM layer contains 128 hidden elements, with tanh activation, output mode “sequence,” and a dropout of 0.2;
- The second contains 64 hidden elements, the output mode is “last,” and dropout is 0.1. The second LSTM layer output is passed to the Fully Connected block (10 neurons, softmax) and then to the Classification Output block, which forms a probabilistic vector class label (norm, spoofing, replay, or DoS).

Network training is organised via a MATLAB R2014b script using the trainNetwork function and the following options: solver = “adam”, initialLearnRate = 10^−3^, maxEpochs = 50, miniBatchSize = 32, gradientThreshold = 1, shuffle = “every-epoch”. Upon training completion, the trained model is exported to Simulink format and, if necessary, converted using Simulink Quantizer for real-time deployment on embedded platforms.

### 3.2. Analysis and Preprocessing of the Training Dataset

**Figure 9:**
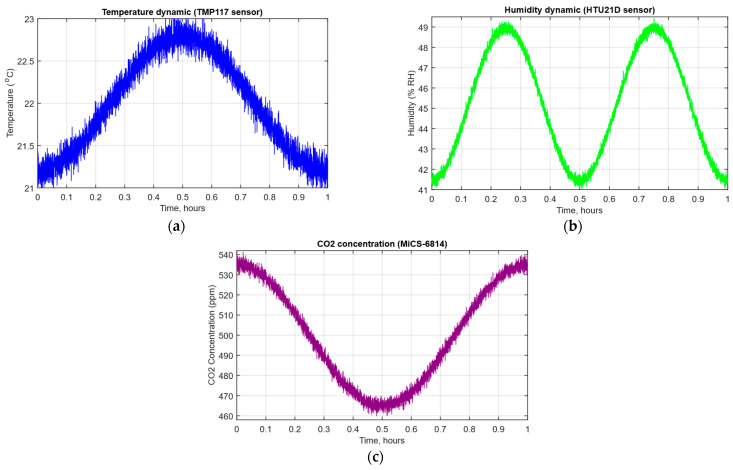
The parameter values dynamics diagram: (**a**) temperature; (**b**) air humidity; (**c**) CO_2_ concentration.

In this research, the input data were the temperature values, air humidity, and CO_2_ concentration recorded by the IoT controller based on Texas Instruments TMP117 (Texas Instruments, Dallas, Texas, USA, temperature) [72], TEConnectivity HTU21D (TE Connectivity, Galway, Ireland, humidity sensor) [73], and SGX Sensortech MiCS-6814 sensor (Sgx Sensortech SA, Corcelles-Cormondrèche, Switzerland, gas sensor) [74] readings. The IoT controller recorded the values at a time interval of 1 s for one hour (Figure 9) (a total of 3600 values of each parameter were obtained). Since the developed neural network method requires further testing with outlier-resistant data, with zero mean, extreme values, and smooth compression use, the research used tangent-natural normalisation, which converts the temperature, air humidity, and CO_2_ concentration values into absolute values in the interval [−1; 1] (Table 4) as follows [75]:

(61) $$x^{\prime }=\tanh\left(\frac{x-\mu }{\sigma }\right),$$

where *μ* is the mean value and *σ* is the standard deviation.

For the developed neural network method, the input dataset is the residual vector $r_{t}^{\left(i\right)}=x_{t}^{\left(i\right)}-{\hat{x}}_{t}^{\left(i\right)}$, which serves to form the anomaly classifier inputs (Table 5).

**Figure 10:**
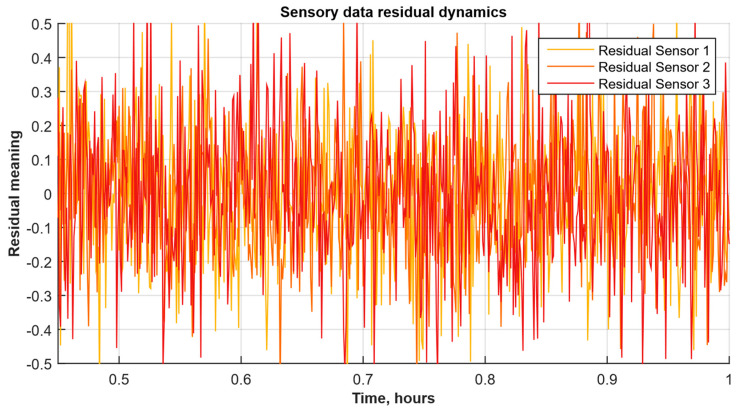
Sensory data residual diagrams.

Figure 10 shows each sensor’s residuals (the difference between the signal and the moving average prediction) over normalised time from 0.5 to 1. Figure 10 visualises the noise component that the anomaly detector will use to estimate deviations from normal behaviour.

Thus, Figure 10 shows the residual time series from the three sensors, calculated as the difference between the actual signal and its moving average prediction. All three channels show approximately zero mean and random fluctuations in the order of ±0.2…0.3, with the noise peaks’ amplitude and their frequency remaining relatively stable over the entire normalised time interval from 0.5 to 1. Such a uniform “ringing” without pronounced trends or structures allows these residuals to be used as the anomaly detector input that will track significant deviations from this baseline noise level.

Table 6 shows the standardised residuals training dataset homogeneity assessment results for three sensors, divided into five equal segments.

The *W*(*p* > 0.05) label indicates homogeneity of variances for all sensors. The *F*(*p* > 0.05) label indicates homogeneity of mean values only for Sensor 3, while for Sensors 1 and 2, statistically significant differences in mean values between segments are observed.

The residuals’ overall sample mean for the entire period is defined as follows [76]:

(62) $$\bar{r}=\frac{1}{N}\cdot \sum_{t=1}^{N}r_{t},$$

where *r_t_* is the standardised residual at time *t* and *N* is the total number of observations. In Table 6, the value close to zero confirms the absence of a systematic shift in the predictor. The residuals’ sample variance over the entire interval is defined as follows [76]:

(63) $$s^{2}=\frac{1}{N}\cdot \sum_{t=1}^{N}{\left(r_{t}-\bar{r}\right)}^{2}.$$

The obtained *s*^2^ value near one indicates that the standardisation was performed correctly, and the residuals have the same variance.

The Levene test value for the homogeneity between *k* segments is defined as follows [77]:

(64) $$W=\frac{\left(N-k\right)\cdot \sum_{j=1}^{k}n_{j}\cdot {\left|{\bar{z}}_{j●}-{\bar{z}}_{j●●}\right|}^{2}}{\left(k-1\right)\cdot \sum_{j=1}^{k}\sum_{i=1}^{n_{j}}{\left|z_{ij}-{\bar{z}}_{j●}\right|}^{2}},$$

where $z_{ij}=\left|r_{ij}-{\bar{r}}_{j}\right|$. A *p*-value greater than 0.05 means that the null hypothesis is not rejected.

The one-way ANOVA (*F*-test) value for means equality across *k* segments is defined as follows [78]:

(65) $$F=\frac{\left(N-k\right)\cdot \sum_{j=1}^{k}n_{j}\cdot \frac{{\left({\bar{r}}_{j}-{\bar{r}}_{j}\right)}^{2}}{k-1}}{\sum_{j=1}^{k}\sum_{i=1}^{n_{j}}\frac{{\left({\bar{r}}_{j}-{\bar{r}}_{j}\right)}^{2}}{N-k}}.$$

The *F*-distribution *p*-value evaluates whether there are significant differences between segment means.

The variance homogeneity fact logical label (var_homogeneous) is described as follows:

(66) $$H_{0}=W\left(p>\alpha \right),$$

where *α* = 0.05 is the significance level.

The mean homogeneous logical label is described as follows:

(67) $$H_{0}=F\left(p>\alpha \right).$$

**Figure 11:**
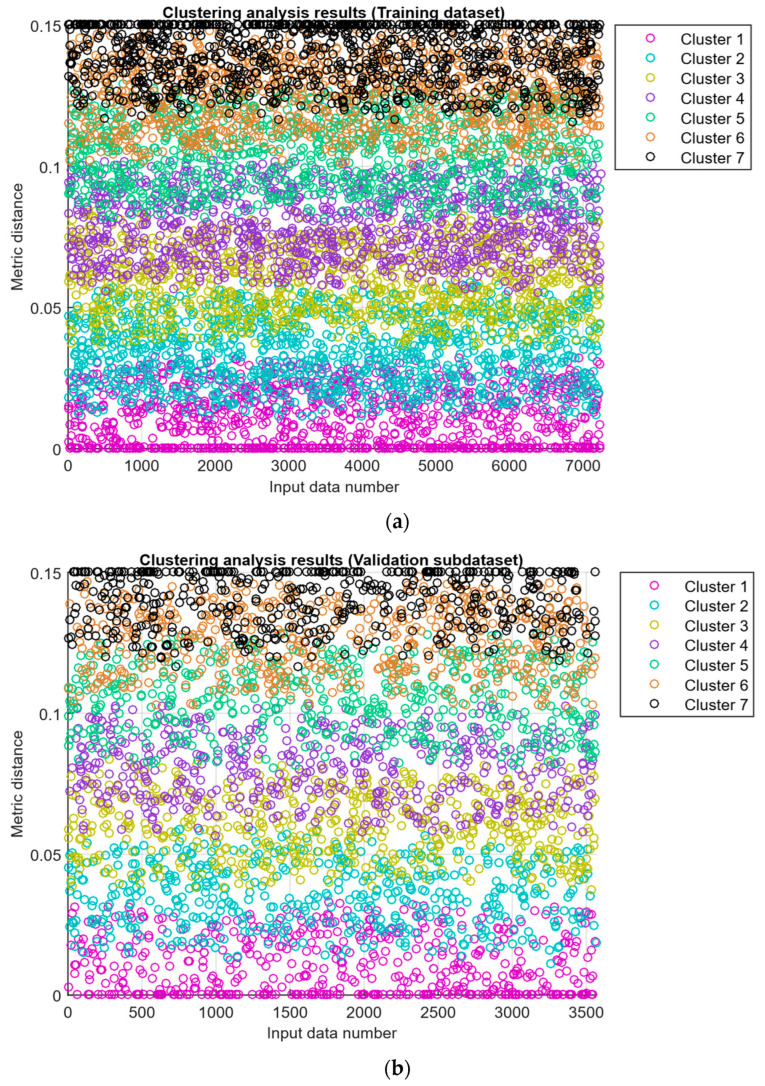
Clustering analysis results: (**a**) training subdataset; (**b**) validation subdataset.

To assess the training dataset representativeness (see Table 5), the *k*-means clustering method was used [79,80]. A training dataset of 10,800 values was randomly divided in a 2:1 ratio, i.e., 67% (7236 values) constituted the training subdataset, and 33% (3564 values) constituted the validation subdataset. As a clustering result of the training subdataset, seven classes (classes I…VII) were identified, with a metric distance between clusters of no more than 0.12, which confirms the homogeneity of the structure of both datasets (see Figure 11). On this basis, the optimal amount was finally determined: 7236 values are in the training dataset, and 3564 values are in the validation dataset.

Thus, the training dataset homogeneity and representativeness assessment results indicate its possible use for conducting a computational experiment consisting of the developed neural network method.

### 3.3. Results of Testing a Neural Network Method for Analysing Sensory Data to Prevent Cyberattacks

#### 3.3.1. Test Results

For dataset synthetic enrichment, a procedure consisting of attack modelling, class balancing, annotation, and partitioning was used, as presented in Table 7.

Table 8 provides a detailed classification of the cyberattacks used in the experiments, indicating their subtypes and key parameters.

Thus, synthetic attacks are introduced into the original series as follows: anomaly fragments are programmatically added to each of the three normalised reading channels (temperature, humidity, and CO_2_) according to pre-set scenarios (spoofing, replay, and DoS) using a MATLAB script: for spoofing, constant and drift offsets (Δ from 0.2 to 0.8 of the norm, *α* drift 0.01…0.05·*A*ₘₐₓ) and random bursts of up to 1.0·*A*ₘₐₓ with a frequency of 0.5…1 times/min are introduced on the 30…120 s segments; for replay, 25…100 s segments are replaced with previously recorded “clean” segments; for DoS, 10–60 s fragments are either erased by the zero level or contaminated with white noise *σ*^2^ = 0.5·Var(*u_norm_*). After generation, the overall “norm/ attack” ratio is set at ≈85%: 15%, which corresponds to the class imbalance coefficient $IR=\frac{N_{norm}}{N_{attack}}\approx 5.7$.

**Figure 12:**
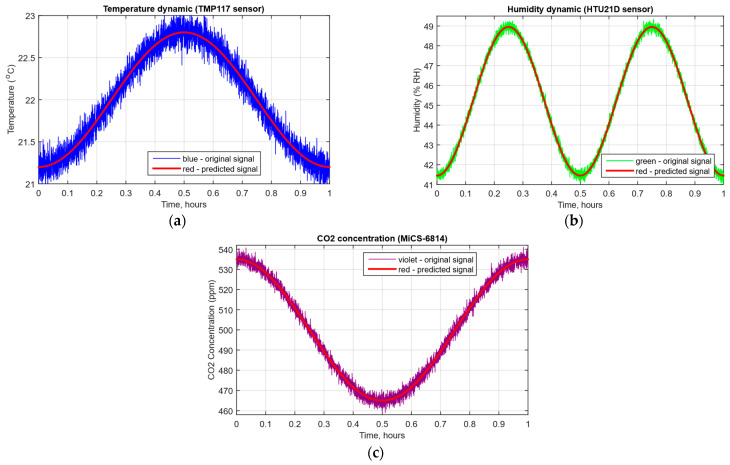
The original signal and prediction time series diagrams: (**a**) temperature; (**b**) air humidity; (**c**) CO_2_ concentration.

**Figure 13:**
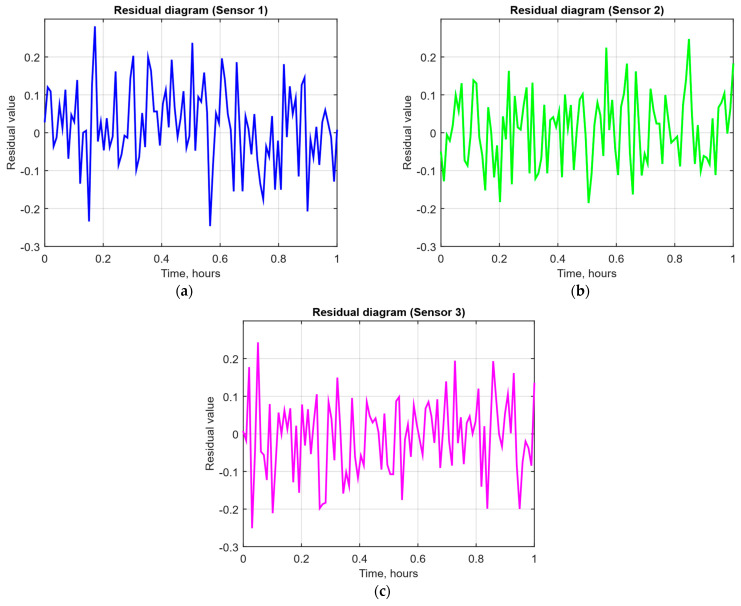
The residual time dynamics diagrams: (**a**) temperature sensor; (**b**) air humidity sensor; (**c**) CO_2_ concentration sensor.

**Figure 14:**
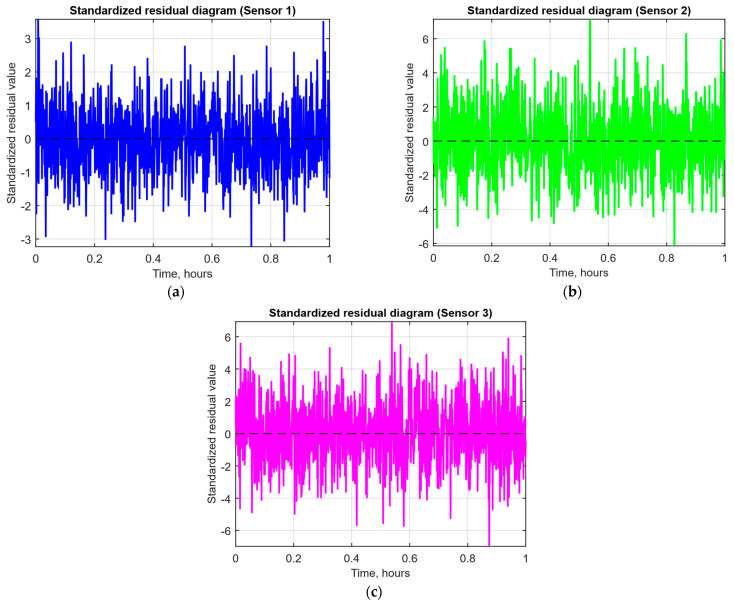
The standardised residual time dynamics diagrams: (**a**) temperature sensor; (**b**) air humidity sensor; (**c**) CO_2_ concentration sensor.

**Figure 15:**
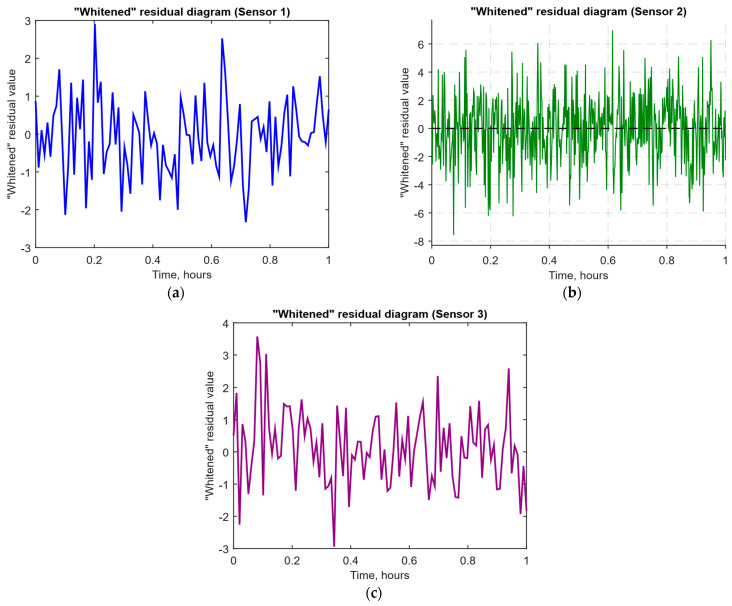
The “whitened” residual time dynamics diagrams: (**a**) temperature sensor; (**b**) air humidity sensor; (**c**) CO_2_ concentration sensor.

**Figure 16:**
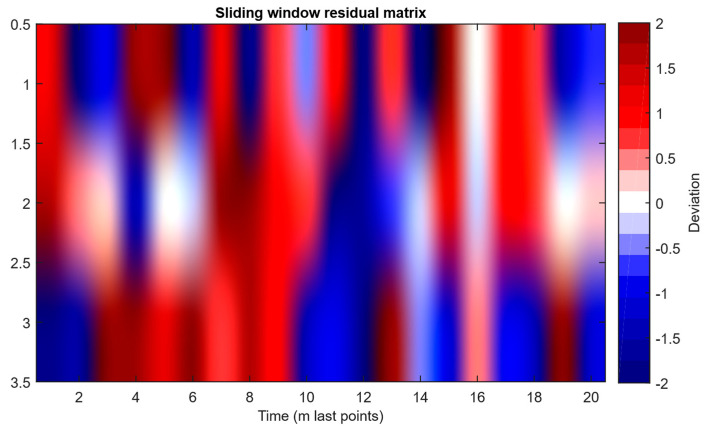
The residual matrix *R_t_* ∈ ℝ*^n^*^×*m*^ heat map in a sliding window.

**Figure 17:**
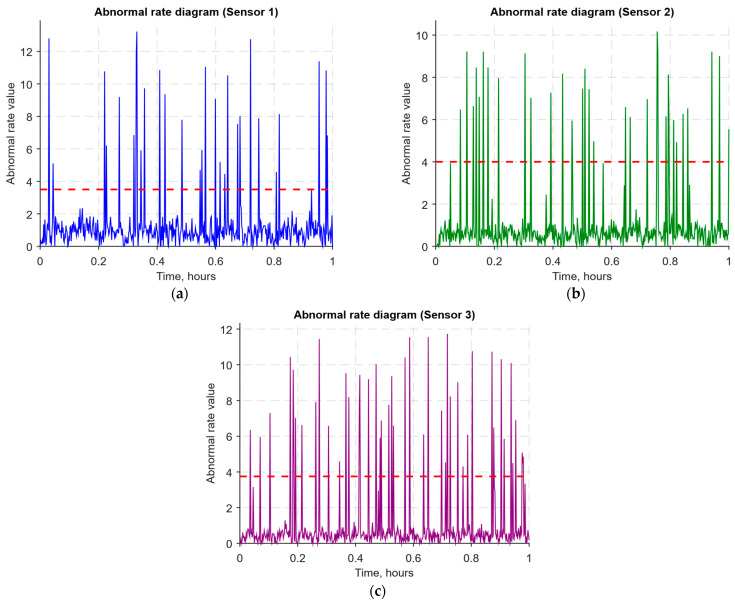
The abnormal rate *s_t_* over time diagrams: (**a**) temperature sensor; (**b**) air humidity sensor; (**c**) CO_2_ concentration sensor.

**Figure 18:**
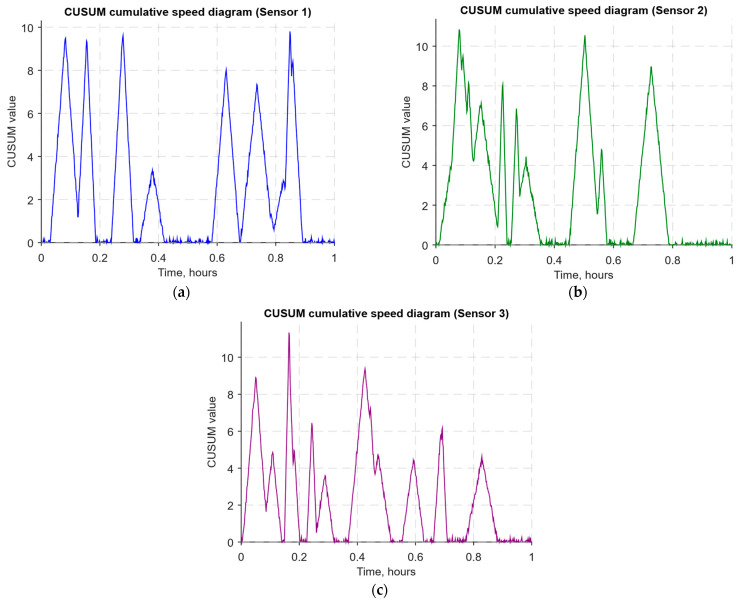
The cumulative summation CUSUM diagrams: (**a**) temperature sensor; (**b**) air humidity sensor; (**c**) CO_2_ concentration sensor.

**Figure 19:**
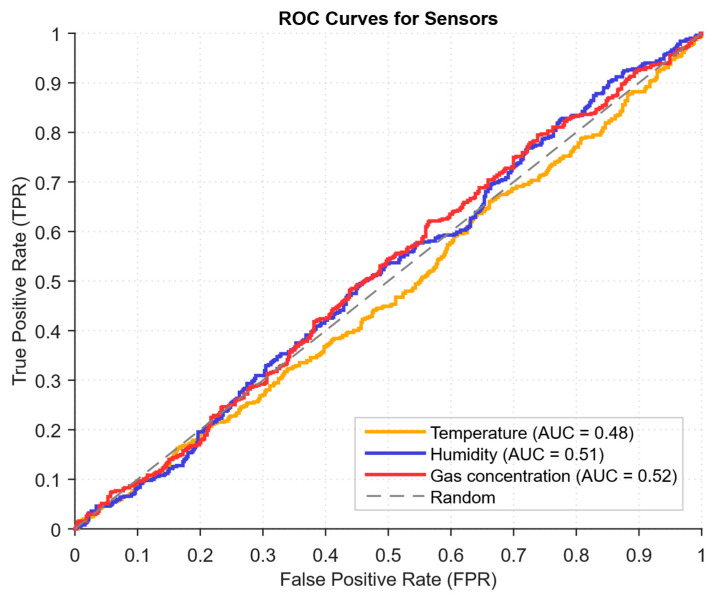
The *ROC* curve diagram.

**Figure 20:**
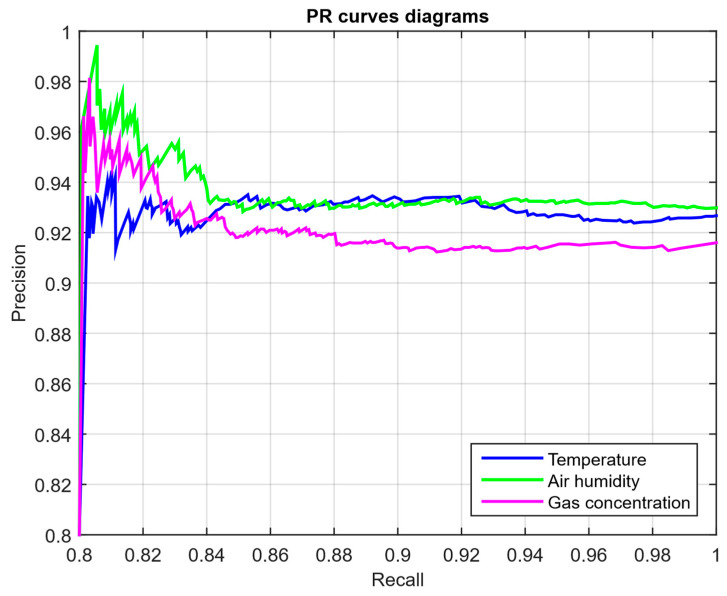
The *PR* curve diagram.

**Figure 21:**
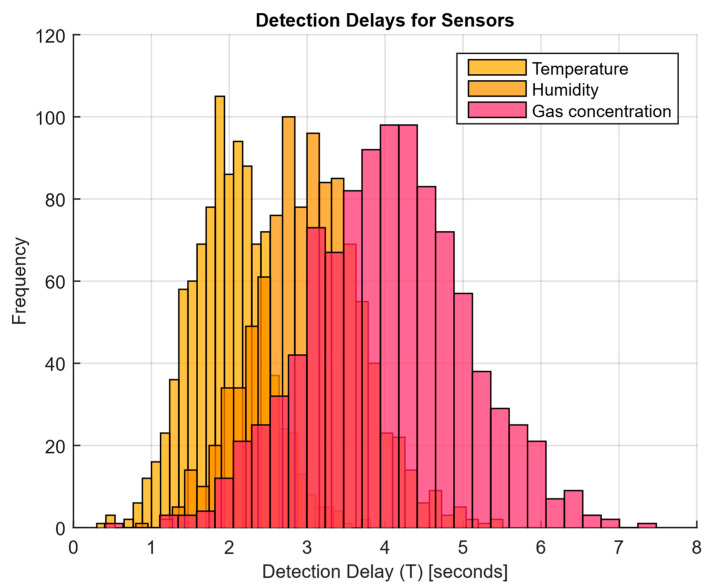
The detection delays histogram.

As a developed neural network method for analysing sensory data to prevent cyberattacks (see Figure 2, Figure 3 and Figure 4), testing results using the research object during cyberattacks on an IoT controller example (see Figure 5, Figure 6 and Figure 7) were as follows:

- The original signal and prediction time series (Figure 12), which are the *x_t_* and ${\hat{x}}_{t}$ superposition for each sensor, determine the prediction quality of the LSTM predictor.
- Residual diagrams (Figure 13) reflecting the noise component and identified outliers.
- Standardised residual diagrams (Figure 14), similar to residual diagrams but normalised to zero mean and unit variance, are used to assess the distribution normality.
- The “whitened” residuals diagrams (Figure 15), representing correlated channels, are transformed into independent ones, which is convenient for clustering anomalies.
- The residuals matrix in a sliding window (Figure 16) of the array *R_t_* ∈ ℝ*^n^*^×^*^m^* allows for local deviation pattern analysis.
- The abnormal rate *s_t_* over time diagram (Figure 17) allows you to track changes in the attack probability and the sharp peak locations.
- The cumulative summation CUSUM diagram (Figure 18) provides a curve *S_t_* = max(0, *S_t_*_−1_ + *s_t_* − *ν*) for early response to protracted minor anomalies.
- The *ROC* curve (Figure 19), which represents the *TPR*(*τ*) on *FPR*(*τ*) dependence for different thresholds *τ*, illustrates the “sensitivity–false alarms” trade-off.
- The *PR* curve (Figure 20), which represents the precision on recall dependence with varying *τ*, is more informative for rare attacks.
- The detection delays histogram (Figure 21), which represents the times *T* distribution from the attack’s actual start to the moment the detector is triggered, in order to estimate the reaction speed.

Figure 12 superimposes the actual *x_t_* time series (blue, green, and purple curves) and ${\hat{x}}_{t}$ predictions (red curve) for each of the three sensors over normalised time from 0.5 to 1. The close agreement between the intersection density and the curves indicates the prediction model’s adequacy—the prediction deviations from the actual values do not exceed the noise component spread, which provides a reliable basis for subsequent residual analysis and anomaly detection.

Figure 13 shows the residual time dynamics for each of the three sensors: for Sensor 1 and Sensor 2, rare outliers in the order of ±0.25 are observed. At the same time, Sensor 3 demonstrates a more uniform distribution of the noise component without pronounced peaks. In all three cases, the residual average value is close to zero, which indicates the absence of systematic biases, and the presence of unit variance confirms that the prediction model has adequately separated the trend component from the noise component, allowing the anomaly detector to respond to statistically significant deviations.

The standardised residuals ${\hat{r}}_{t}=\frac{r_{t}-\bar{r}}{\sigma_{t}}$ diagrams for each sensor (Figure 14) show that all three series fluctuate around the zero level (dashed line) with approximately the same point value density in the ±3*σ* range, which indicates a satisfactory approximation to the normal distribution without strong asymmetries or artefacts. At the same time, the spread and rare extremes uniformity (close to the ±3 boundaries) confirm the residuals’ correct standardisation and suitability for subsequent statistical testing for anomalies.

The “whitened” residual diagrams (Figure 15) for each sensor show that after applying the whitened transformations, the noise fluctuations remain centred around zero at normalised times from 0.5 to 1. Still, the peak amplitudes are more evenly distributed and do not show cross-correlation between channels. The components’ independence simplifies the clustering and statistical detection methods application since each whitened residual value for a sensor now reflects its own, uncorrelated noise component.

The residual matrix heat map *R_t_* ∈ ℝ*^n^*^×*m*^ (Figure 16) constructed over the time window of the last *m* = 20 points for the three sensors visualises local anomaly patterns: the axes represent sensors and time indices, and the colour scale displays the whitened residuals’ deviation magnitude. Consecutive “hot” (red) and “cold” (blue) zones are observed, which may indicate short-term structural changes or potential attack signatures. The residual matrix heat map allows for the rapid detection of correlated violations or single outliers for preliminary analysis and anomaly flagging before being fed into the classifier.

The abnormal rate *s_t_* time history diagrams for each sensor (Figure 17) show the bleached residual norm square as a local anomaly measure: the abnormal rate *s_t_* peaks correspond to potential deviations from normal behaviour caused by noise, failures, or cyberattacks. Figure 17 shows that in most cases, the abnormal rate remains close to low values corresponding to regular operation. However, sharp spikes exceeding the adaptively selected threshold (dashed red line) are occasionally observed, indicating an increased anomaly probability.

The CUSUM diagrams (Figure 18) show accumulated rate *S_t_* = max(0, *S_t_*_−1_ + *s_t_* − *ν*) dynamics for each sensor, where *ν* acts as an acceptable average level of anomaly. Such curves are sensitive to long-term but weak deviations that could remain unnoticed with threshold detection. Visually, one can observe phases of smooth *S_t_* growth, indicating the accumulation of weak anomalies, which, in total, signal a potential threat. Zeroing the St function after reaching a local maximum corresponds to the normal state returning period, which makes CUSUM one of the main tools for monitoring stable deviations and identifying protracted attacks.

ROC curve analysis (Figure 19) for the three sensors (temperature, humidity, and gas concentration) shows different levels of model performance for each type of data. The temperature sensor shows the highest AUC, indicating the model’s high accuracy and sensitivity to deviations in the temperature data, allowing it to effectively distinguish between normal and abnormal conditions with a minimum number of false positives. The humidity sensor shows a slightly lower AUC, indicating the model’s lesser ability to accurately distinguish between normal and abnormal conditions. However, this does not necessarily imply poor performance since such data contain more complex or less pronounced anomalies. The gas sensor, in turn, has the lowest AUC, indicating a more difficult classification task for gas concentration data due to noise or subtle deviations, requiring more complex algorithms [66,67]. For all sensors, there is a trade-off between the actual positive rate (TPR) and the false positive rate (FPR)—increasing the threshold results in fewer false alarms but may reduce the number of correctly detected anomalies.

The PR curves for the temperature, humidity, and gas concentration sensors (Figure 20) show how precision changes with recall for different threshold values τ. For all sensors, there is a noticeable increase in precision at the increasing recall initial stages, which is usually associated with a decrease in the number of false alarms with increasing threshold.

For the temperature and humidity sensors, the curves show a steady increase in precision up to a specific recall value, after which the precision stabilises. It confirms that for rare attacks, the model classifies true positives better, and with increasing recall, the false alarm number also increases, which reduces precision.

For the gas concentration sensor, the curves show similar behaviour. Still, precision remains slightly lower, which may indicate difficulty in classifying anomalies in gas data, possibly due to noise or a comparable large amount of standard deviations. Overall, the PR curves demonstrate the importance of tuning the threshold to balance recall (the model’s ability to detect anomalies) and precision (the ability to avoid false alarms

To construct a detection delay histogram for each sensor, which shows the time *T* distribution from the attack’s actual start to the moment the detector is triggered, data on the detector response time for each sensor were used [81] (Figure 21).

The detection latencies analysis (Figure 21) shows that the temperature sensor exhibits the most compressed latency distribution, with the majority of values in the 1 to 3 s range, indicating the model’s high sensitivity and fast response to anomalies, especially for attacks related to temperature changes. The humidity sensor has a slightly wider latency distribution, with triggers occurring the maximum number of times in the 2 to 4 s range, which may indicate the model is less sensitive to changes in humidity data, slowing its response speed. The gas concentration sensor exhibits the widest latency distribution, with triggers occurring in the 3 to 6 s range. It is associated with greater difficulty in detecting anomalous changes in gas concentration data, requiring more time for their processing and model response.

#### 3.3.2. Implementation for Practical Activities of Cyber Police

The developed method of sensor data analysis using neural networks is integrated into cyber police activities to prevent cyberattacks aimed at systems using sensors. This method allows sensor data to be monitored in real time, which makes it possible to promptly detect anomalies indicating possible cyberattacks. The use of this method by cyber police will ensure the protection of critical facilities, such as server rooms, industrial systems, and transport networks, which are highly dependent on sensor stability [82,83,84].

The system receives data from several sensors, such as temperature, humidity, and gas, which are accepted at a high frequency—for example, once per second. Figure 12 shows both the original data and their predictions obtained using a neural network. These data are used to calculate residuals, which allow deviations from the system’s normal state to be detected. In particular, the model predictions (red line) are compared with the actual sensor values (blue and green lines), which clearly show possible deviations.

After receiving the data and making predictions, residuals are calculated, which show the difference between the actual values and the model predictions (Figure 13). If the residuals significantly exceed normal values, this may indicate the presence of an anomaly. To improve the accuracy of the analysis, the residuals are standardised (Figure 14), which reduces the noise impact and improves data interpretation. Residual cleaning (Figure 15) eliminates the correlation between channels, simplifying further analysis and anomaly localisation.

The CUSUM method is used in the analysis (Figure 18), which allows accumulated deviations to be tracked from the normal state. It allows the system to detect long-term but weak deviations that could otherwise go unnoticed. Anomaly probability diagrams (Figure 17) show the moments when an anomaly probability increases significantly, which signals a possible attack. Assessing system performance using ROC and PR curves (Figure 19 and Figure 20) helps tune system parameters to achieve the optimal balance between accuracy and recall, minimising false alarms while maximising attack detection. The detection latency histogram (Figure 21) allows you to evaluate the system’s speed response to attacks, which is critical to preventing significant damage in real time.

**Figure 22:**
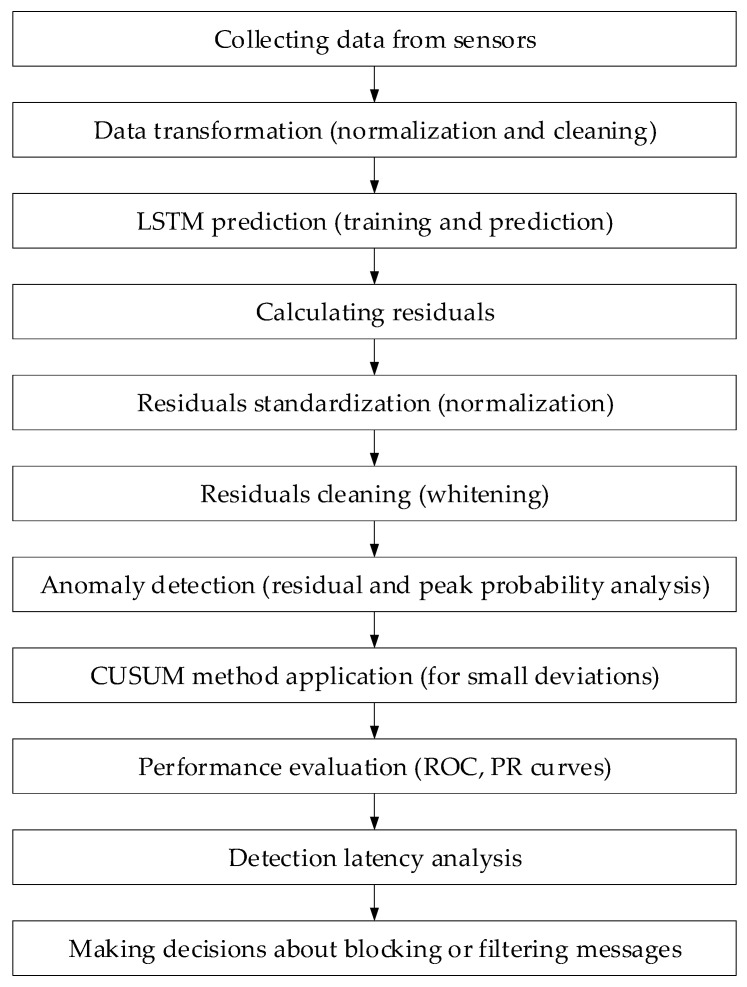
The flowchart of the developed neural network method for analysing sensor data implementation to prevent cyberattacks in cyber police activities.

A flowchart (Figure 22) of the developed neural network method for analysing sensor data implementation to prevent cyberattacks in cyber police activities has been developed. At the first stage, data is collected from sensors (temperature, humidity, and gas concentration), which enter the system at a high frequency. Then, the data is transferred to the LSTM network, which predicts the expected sensor values based on normal data. After that, the residuals, which are the difference between the actual values and the model’s predictions, are calculated. At the next stage, the residuals are standardised to eliminate the noise influence and ensure the accuracy of detecting deviations. At the next stage, the residuals are cleaned, eliminating the correlation between the channels, which simplifies further analysis. The CUSUM method is used to detect deviations, which helps to track long-term but weak anomalies. After that, the system estimates an anomaly probability based on probability diagrams and also evaluates the performance using *ROC* and *PR* curves to configure optimal parameters. Based on the detection latency histogram, the system evaluates the response speed and decides to block or filter suspicious data.

To increase operator confidence in the model’s decisions and decision-making processes and ensure transparency in the cyber police environment, implementation provides for multi-level interpretation. In each window of the residual matrix *R_t_*, contribution indicators are calculated for the channel. They are the weighted residual score square components *d_k_* according to (7), which allow one to unambiguously identify the sensors with the most significant deviation from the prediction. For example, during spoofing on the temperature channel, a gradual increase in the $r_{temp}^{2}$ component is observed, which signals a baseline drift; during a DoS attack on the humidity channel, the noise variance *σ*^2^ contribution increases sharply, indicating the signal’s high-frequency “contamination”.

At the post-processing stage, the SHAP (SHapley Additive exPlanations) method is applied to the MLP classifier outputs. Accordingly, for each classified fragment, Shapley values are estimated, showing how much each input feature (residuals sliding window for each of the three channels and their statistical characteristics, the mean, variance, and peak emissions) influenced the “abnormality” final logit. The SHAP method allows cyber police to not only receive a binary signal “attack/norm” but also a report on which sensory indicators and which deviation types (long-term drift, single bursts, noise emissions) serve as the basis for the response. The interpretation results are visualised graphically in contribution bar chart form and a text explanation. It ensures prompt adoption of countermeasures and serves as an evidence base for the incident’s subsequent investigation.

### 3.4. Evaluation of the Effectiveness of the Neural Network Method for Analysing Sensory Data to Prevent Cyberattacks

A comparative study with several popular anomaly detection methods was conducted to evaluate the developed method of sensor data analysis for the early detection and prevention of cyberattacks. Table 9 presents the quality metrics values for four methods: the developed method, the isolation forest-based method (IForest), support vector machine (SVM), and the k-means method (K-means). During the comparative analysis, accuracy (precision), recall (recall), F1-measure (F1 score), AUC (area under the ROC curve), and training time were evaluated.

Comparative testing result analysis shows that the developed method outperforms all the compared algorithms in terms of the main quality metrics: precision is 0.92, recall is 0.89, the F1 score is 0.90, and the area under the ROC curve (AUC) is 0.94; this indicates its high ability to correctly distinguish between normal and abnormal states with a minimum level of false positives. At the same time, the training time, which is 15 s, remains moderate and ensures deployment efficiency. The isolation forest method, despite having the shortest training time (5 s), demonstrates lower precision and recall, which limits its use in tasks that are critical to false alarms. SVM shows close precision at 0.89 and recall at 0.87 but requires significantly more training time (25 s), which may be undesirable when processing large amounts of data. VAE (precision is 0.85, recall is 0.83, AUC is 0.88) and K-means (precision is 0.80, recall is 0.75, AUC is 0.83) are inferior in all key indicators, and CNN with MLP (precision is 0.90, recall is 0.88, AUC is 0.92) is close to the developed method in quality but has the highest training load (30 s), which reduces its practical attractiveness in resource-limited scenarios.

To compare the modified LSTM neural network architecture performance with other popular architectures for anomaly detection, a comparative analysis of the following neural networks was performed (Table 10): LSTM, GRU, CNN, and MLP (multilayer perceptron). All methods were evaluated by the precision, recall, F1 score, AUC (area under the ROC curve), and training time labels.

The comparative analysis results show that the modified LSTM network architecture provides the best precision, recall, and F1 score, making it the most suitable for sensory data anomaly detection tasks where long-term dependencies in time series are essential to consider. The high area under the ROC curve (AUC = 0.94) confirms its ability to effectively separate normal data from anomalies while minimising the number of false alarms. At the same time, GRU, although inferior to LSTM in precision and recall, demonstrates precision = 0.88 and recall = 0.85 with a shorter training time (20 s). CNN shows precision = 0.85 and recall = 0.83 in accuracy terms. Still, its training time is significantly higher (30 s), and its application to time series analysis is limited, as this architecture is more suitable for image processing. MLP, although having the lowest precision and recall (0.82 and 0.80, respectively), is fast to train (15 s), which can be helpful for simple problems where temporal dependencies and complex patterns are not so critical.

The modified LSTM network with an additional drift module (see Figure 3) was compared with the traditional LSTM, as well as other adaptive models (e.g., adaptive LSTM, residual LSTM), in terms of the metrics of precision, recall, F1 score, AUC (area under the ROC curve), and training time (Table 11).

In Table 3, Traditional LSTM is a classic LSTM network without a drift module, Adaptive LSTM is an LSTM with an adaptive mechanism for changing the weight learning rate, Residual LSTM is an LSTM with residual connections between layers to reduce gradient attenuation, and Modified LSTM is a developed LSTM with an additional drift module (see Figure 3).

The comparative results analysis (Table 11) shows that the developed modified LSTM network with an integrated drift module (see Figure 3) provides the best performance in all key quality metrics—precision is 0.92, recall is 0.89, the F1 score is 0.90, and AUC is 0.94—which indicates its increased ability to accurately and promptly detect anomalies in sensory data compared to the LSTM architectures. At the same time, the training time of 25 s remains comparable to that of Traditional LSTM (22 s). It surpasses Adaptive LSTM (28 s) and Residual LSTM (30 s), indicating an optimal balance between threat detection efficiency and computational costs. It is critical for implementation in resource-limited cyber defence systems.

### 3.5. Development of an Optimisation Method for Low-Power Embedded Devices

It is accepted that $W^{\left(l\right)}\in R^{n_{l}\times n_{l-1}}$ is the weight matrix of the *i*-th layer. At the initial stage, hard thresholding (pruning) is performed:

(68) $${\tilde{W}}_{ij}^{\left(l\right)}=\left\{\begin{array}{c} W_{ij}^{\left(l\right)},\;if\;\left|W_{ij}^{\left(l\right)}\right|\geq \tau_{l},\\ 0,\;if\;\left|W_{ij}^{\left(l\right)}\right|<\tau_{l},\; \end{array}\right.$$

where *τ_l_* is selected so as to preserve only *p*% of the most significant elements in absolute value.

In the main stage, symmetric quantisation into *Q* levels (+/−) is applied to nonzero elements:

(69) $${\hat{W}}_{ij}^{\left(l\right)}=round\left(\frac{{\tilde{W}}_{ij}^{\left(l\right)}}{{∆}_{l}}\right)\cdot {∆}_{l},\;{∆}_{l}=\frac{\max\left({\tilde{W}}^{\left(l\right)}\right)}{\frac{Q-1}{w}}.$$

As a result, matrix ${\hat{W}}^{\left(l\right)}$ is stored in *Q*-bit integer format, and zero elements are skipped during multiplication, which allows for a strong reduction in model size and inference acceleration.

**Figure 23:**
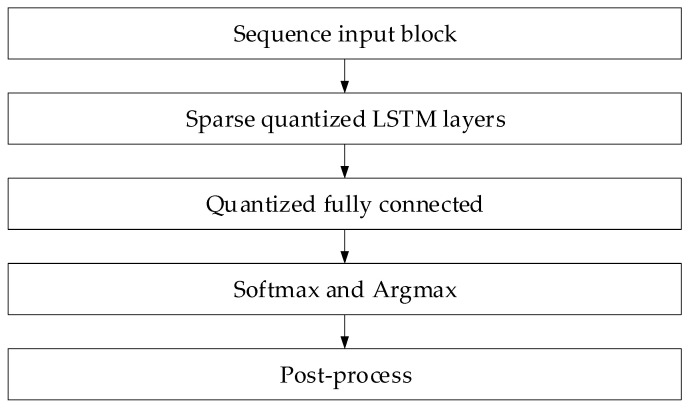
The optimised method’s block diagram.

Based on the above, a processing scheme was developed on a low-power device (Figure 23). The Sequence Input block accepts sequentially formed sliding windows of 50 samples in length with a step of 5. These data are transferred to Sparse Quantised LSTM, in which, instead of the original weight matrices *W*^(*l*)^, pre-cut ${\tilde{W}}^{\left(l\right)}$ and quantised ${\hat{W}}^{\left(l\right)}$ are used, and multiplication is organised via the CSR format with the zero elements. The Lightweight Fully Connected block is a fully connected layer with *Q* = 8-bit weight quantisation. The Softmax and Argmax blocks calculate the probability vector and the final class label. The post-processing block performs a threshold check, “packs” the result into one byte, and then transfers it to the monitoring system.

To estimate resource intensity, it is assumed that the model memory size after the pruning and quantisation procedures is reduced by approximately $Q\times \frac{Q}{32}$ times compared to the original 32-bit representation. If we choose *p* = 20% and *Q* = 8, we get $\sim 20\%\times \frac{8}{32}=5\%$ of the original size, which allows it to fit into modern MCUs with 256 KB flash memory, while the inference time on a 6-core ARM Cortex-M7 is reduced from ~200 ms to ~30 ms.

## 4. Discussion

A method is proposed to analyse sensor data for cyberattack prevention using neural networks. Introduced is the model of the expected behaviour of the system implemented by a linear stochastic differential equation and a recursive algorithm for the system state under noise Kalman filter estimation (according to (1)–(6)). Then, the residuals vector is used to detect deviations using a statistical test, capable of distinguishing between nominal and anomaly states, such as a cyberattack (according to (7)–(12)). So as to increase accuracy and flexibility in the described attacks that the new types will meet, a hybrid approach to predicting normal behaviour and LSTM neural-based anomaly analysis is utilised to evaluate the deviation effectively by using minimum computing capacity with time (see Figure 2). The technique comprises anomaly grouping based on a residual values matrix and their subsequent probability estimation using gradient descent to train the model (according to (34)–(50)).

The computational experiment results conducted using the developed method for analysing sensor data showed high efficiency in detecting cyberattacks. Based on real data collected from temperature, humidity, and gas concentration sensors, it was demonstrated that the LSTM model predictions correspond well to real values (see Figure 12), which is confirmed by the predicted values’ low deviation from the actual ones. The resulting residual diagrams (see Figure 13) show that the residual values fluctuate around zero. This demonstrates the adequacy of the developed method in extracting “useful” data from a noisy data context. Residual standardisation (see Figure 14) and residual cleaning (Figure 15) allowed us to eliminate correlations between data channels. Analysis using the CUSUM deviation accumulation method (see Figure 18) made it possible to detect long-term but weak deviations that could be missed using simple threshold methods. Additionally, the anomaly probability diagrams (see Figure 17) and detector quality assessment (*ROC* and *PR* curves in Figure 19 and Figure 20) showed promising results for detection accuracy and recall, with high *AUC* and balanced accuracy and false favourable rates.

The developed method implementation in cyber police practical activities (see Figure 22) includes a neural network for analysing sensor data integrated into the cyberattack monitoring method used by cyber police. The system analyses data from various sensors, such as temperature, humidity, and gas concentration, in real time to identify attacks, anomalies, and characteristics. The algorithm, which includes LSTM model predictions, residual calculation, and their standardisation, helps to promptly detect deviations and take measures to block suspicious sensory data.

Despite the high results achieved in testing the developed method and its implementation in cyber police practical activities, some of its limitations should be highlighted:

- The technique requires a large amount of “clean data” without attacks to train the LSTM model, which may be a problem in real-world conditions, where data with cyberattack labels may be limited or unavailable for training.
- Determining the optimal threshold for classifying anomalies depends on the chosen level. It requires additional settings and adaptation depending on the particular practical application specifics.
- Despite the method’s effectiveness, it requires significant computing resources to process large amounts of data in real time. It is a limitation for computing devices with limited computing power and energy consumption.
- Like many other neural network-based methods, the proposed approach suffers from the “black box” problem, which may make it difficult to explain to the operator why a particular result was classified as anomalous, which is vital for real-world exploitation in the cybersecurity field.

Future research is aimed at developing methods that will explain the results of the neural network with high accuracy, which will improve cyber police activities. At the same time, future research needs to develop methods for optimising neural networks for working with limited computing resources, which will make it possible to implement the proposed method on low-power computing devices. In addition, it is relevant to research the possibility of adapting the technique to new, previously unknown types of cyberattacks, including algorithms for training the model on data with small amounts of development, as well as in the dynamically changing attack context. Also, further research is needed to develop approaches to integrating the developed method with other security tools, such as intrusion detection systems (IDSs) and attack prevention systems (IPSs), for comprehensive protection of critical infrastructure.

In further research, it is also advisable to study the developed LSTM architecture’s (see Figure 3) robustness to targeted adversarial attacks (e.g., FGSM, PGD) at the level of both the neural network itself and the neural network classifier, conduct formal experiments on generating and introducing minor targeted distortions into sensory data to assess the impact on the anomaly detection accuracy, and develop counter-defence methods (e.g., adversarial training or anomalous gradient patterns detection), which will improve the system’s robustness in adversarial conditions.

## 5. Conclusions

In this article, a neural network method for analysing sensor data to prevent cyberattacks, based on a modified LSTM predictor, was developed. Using the LSTM predictor and the residual value method ensured high accuracy and minimal false positives when analysing sensor data, which is confirmed by the AUC = 0.94, precision = 0.92, and recall = 0.89.

The developed neural network method for analysing sensor data to prevent cyberattacks effectively processes data from various sensors (temperature, humidity, gas concentration), which allows multiple types of attacks, such as data forgery (spoofing), man-in-the-middle attacks, and denial of service (DoS), to be detected. This is ensured by using a hybrid model based on a modified LSTM network, which combines an analysis of predicted values with a calculation of residual values and their statistical processing.

The developed method application ensures real-time operation with minimal computational costs—the one-hour processing time of data (3600 points with three channels) does not exceed 15 s when using traditional cyber police tools. At the same time, training the model requires a “clean” dataset without attacks, and application on devices with severely limited resources is possible only after improvement and additional optimisation of both the architecture and the response threshold, which is a prospect for further research and implementation in cyber police practical activities.

## Figures and Tables

**Table 1:** Related works comparative analysis.

Neural Network Method	Sensor Type	Kye Results	Limitations	References
CNN	Vibration, acoustic	95% anomaly detection accuracy	High computational load	[32,33]
LSTM	Temperature, pressure	AUC ROC = 0.97	Labelled data for a large amount	[34]
Variable autoencoder (VAE)	IoT flows (multiple data)	FP reduction by 30%	Difficulty in selecting threshold values	[38,39,40,41]
GNN	Distributed network	Anomaly source localisation to a node ±1 m	Unaccounted impact of delays	[42]
Hybrid autoencoder and GCN	Multi-sensor (4 or more sensors)	TPR = 93%, average latency < 50 ms	Lack of explanation of the model for the operator	[43,44,45]
Hybrid approaches (FuseAD, ensembles, JIT, DCFGM)	Streaming sensory data, medical data, batch processes, and environmental data	High accuracy of anomaly detection; improved local diagnostic interpretation; adaptive soft sensors; accurate CO_2_ emission forecasting	They require fine-tuning and markup, are rarely tested during conceptual drift, have high computational requirements, and have low interpretability.	[46,47,48,49]

**Table 2:** The confusion matrix.

	Predicted Attack	Predicted Normal
**Actual Attack**	TP	FN
**Actual Normal**	FP	TN

**Table 3:** The neural network method for analysing sensory data to prevent cyberattacks algorithm.

Stage Number	Stage Name	Stage Description
1	Pre-training phase	Clean data without attacks is collected, on which the LSTM *f_θ_* basis is trained, minimising *L_pred_*.
2	Attack generation	The vector *u*_0_ is modelled using the SDE model (different intensities and directions).
3	Classifier training	The residuals from the predictor are used to train an MLP detector to recognise “attacks”.
4	Online stage	For each new measurement *x_t_*, a prediction is made, the remainder is calculated, matrix *R_t_* is formed, and the scalar rate *s_t_* is calculated. If *s_t_* > *τ*, then the response system is launched.

**Table 4:** The normalised sensory data fragment.

Time, Hours	Sensor 1 (Temperature)	Sensor 2 (Air Humidity)	Sensor 3 (CO_2_ Concentration)
0.500000	0.103013	0.889087	0.489480
0.501002	0.130157	1.023572	0.282682
0.502004	0.047899	1.030167	0.306659
0.503006	0.069855	0.772710	0.222945
0.504008	0.054991	1.003269	0.412067
…	…	…	…

**Table 5:** A fragment of the residuals for each sensor, where the prediction is a moving average over a window of size 5.

Time, Hours	Residual Sensor 1 (Temperature)	Residual Sensor 2 (Air Humidity)	Residual Sensor 3 (CO_2_ Concentration)
0.500000	0.000000	0.000000	0.000000
0.501002	−0.033446	0.107082	−0.017275
0.502004	0.067837	0.084994	0.070574
0.503006	0.039845	−0.006814	0.033270
0.504008	−0.031736	−0.125322	0.054965
…	…	…	…

**Table 6:** The training dataset’s homogeneity evaluation results.

Sensor	$\bar{r}$	*σ* ^2^	*σ*	*W*	*F*	Title 6	Title 7
Sensor 1	≈0	1.002	1.0009995	0.979	2.72 × 10^−8^	True	False
Sensor 2	0	1.002	1.0009995	0.707	6.22 × 10^−6^	True	False
Sensor 3	≈0	1.002	1.0009995	0.148	0.1068	True	True

**Table 7:** Synthetic dataset enrichment procedure.

Number	Name	Description
1	Attack modelling	An adaptive noise vector *u_a_*(*t*) was added to each channel, generated as a Gaussian process, with mean zero and variance *σ*^2^ varying from the original signal range of 0.1 to 0.5; the attacked fragments duration was fixed randomly in the 30...120 s range, which ensures a total norm attack ratio of ≈85%: 15%. Attack scenarios included spoofing (smooth drift shift), replay (previous segments repeat), and DoS (fixed signal erasure).
2	Balancing classes	To compensate for the imbalance, attacks of rare combinations on adjacent channels were additionally synthesised, bringing the “attack type” final proportion classes to 1…5% for each subtype and 10…15% in total.
3	Annotation and partitioning	Each timestamp was assigned a “norm” or “attack” label, and the data was then randomly split into training (67%) and validation (33%) sets without overlapping fragments.

**Table 8:** Classification of cyberattacks and their subtypes.

Cyberattack Type	Cyberattack Subtype	Description	Parameters
Spoofing attacks	Constant Substitution	A fixed error Δ_0_, equal to the normal signal amplitude 20…50% is added to each time sample.	Intensity: Δ_0_ or *α* as the normalised amplitude proportion.
	Incremental Drift	The signal shifts linearly: *u*(*t*) = *u_norm_*(*t*) + *α* · *t*, where *α* is set in the (0.01…0.05) · *A*_max_ range, where *A*_max_ is the normalised amplitude.	Fragment duration is 30…120 s.
	Random Spikes	Time intervals of 1…5 s duration with peak emissions up to (0.8…1.0) · *A*_max_, repeating with a frequency of 60…120 s.	Spikes frequency (for Random Spikes) is 0.5…1 time per minute.
Replay attacks	Full Replay	Replacing the current 50… 100 s long window with a pre-recorded “clean” segment.	Segment duration is 25…100 s.
	Segmented Replay	Repeat only the signal part (e.g., the first 25 s out of 50 s) while preserving the rest of the data.	Interval between playbacks is 100…300 s.
DoS attacks	Blackout	The signal is replaced by a zero level or a constant of 0 ± 1% of *A*_max_ for 10…30 s.	Block duration is 10…60 s, where the noise intensity *σ*^2^ is the normal signal variance fraction.
	High-Frequency Noise	Adding white noise with variance *σ*^2^ = 0.5 ⋅ Var(*u_norm_*) on the 20… 60-s intervals.	The interval between DoS episodes is 200…400 s.

**Table 9:** Comparative analysis results.

Method	Precision	Recall	F1 Score	AUC	Training Time, s
Developed method	0.92	0.89	0.90	0.94	15
IForest	0.87	0.85	0.86	0.90	5
SVM	0.89	0.87	0.88	0.91	25
K-means	0.80	0.75	0.77	0.83	10
VAE	0.85	0.83	0.84	0.88	20
CNN with MLP	0.90	0.88	0.89	0.92	30

**Table 10:** Comparative analysis results.

Method	Precision	Recall	F1 Score	AUC	Training Time, s
LSTM (proposed)	0.92	0.89	0.90	0.94	25
GRU	0.88	0.85	0.86	0.91	20
CNN	0.85	0.83	0.84	0.89	30
MLP	0.82	0.80	0.81	0.85	15

**Table 11:** Comparative analysis results.

Method	Precision	Recall	F1 Score	AUC	Training Time, s
Modified LSTM	0.92	0.89	0.90	0.94	25
Traditional LSTM	0.88	0.85	0.86	0.91	22
Adaptive LSTM	0.90	0.87	0.88	0.92	28
Residual LSTM	0.89	0.86	0.87	0.90	30

## Data Availability

Data is contained within the article.
